# Supplementary material for: Quality and outcomes in global cancer surgery: protocol for a multicentre, international, prospective cohort study (GlobalSurg 3)
Source: BMJ Open. 2019 May 24;9(5):e026646. doi: 10.1136/bmjopen-2018-026646 (PMC6538014; doi:10.1136/bmjopen-2018-026646)
Supplement: Supplementary file 5 [file bmjopen-2018-026646supp005.pdf]

South East Scotland Research Ethics Service

Waverley Gate
2-4 Waterloo Place
Edinburgh
EH1 3EG

NHS
Lothian

Ewen M Harrison

Senior Lecturer in General Surgery /
Honorary Consultant Surgeon
NIHR Unit on Global Surgery (Universities of Birmingham, Edinburgh and Warwick)
Clinical Surgery
University of Edinburgh
Royal Infirmary of Edinburgh
Edinburgh
EH16 4SA

Date: 19/02/2018
Your Ref:
Our Ref: NR/161AB6
Enquiries to:
Direct Line: 0131 465 5679
Email:

Dear Mr Harrison,

Project Title: "GlobalSurg 3: Quality and outcomes in global cancer surgery: a prospective, international cohort study"

You have sought advice from the South East Scotland Research Ethics Service on the above project. This has been considered by the Scientific Officer and you are advised that, based on the email correspondence it does not need NHS ethical review under the terms of the Governance Arrangements for Research Ethics Committees (A Harmonised Edition).

If the project is considered to be health-related research you will require a sponsor and ethical approval as outlined in The Research Governance Framework for Health and Community Care. You may wish to contact your employer or professional body to arrange this. You may also require NHS management permission (R&D approval). You should contact the relevant NHS R&D departments to organise this.

For projects that are not research and will be conducted within the NHS you should contact the relevant local clinical governance team who will inform you of the relevant governance procedures required before the project commences.

This letter should not be interpreted as giving a form of ethical approval or any endorsement of the project, but it may be provided to a journal or other body as evidence that NHS ethical approval is not required. However, if you, your sponsor/funder feel that the project requires ethical review by an NHS REC, please write setting out your reasons and we will be pleased to consider further. You should retain a copy of this letter with your project file as evidence that you have sought advice from the South East Scotland Research Ethics Service.

Yours sincerely,

Helen Newbery

Helen Newbery
Scientific Officer
South East Scotland Research Ethics Service

INVESTORS IN PEOPLE
POSITIVE ABOUT DISABLED PEOPLE
Healthy Working Lives

Headquarters
Waverley Gate, 2-4 Waterloo Place
Edinburgh EH1 3EG
Chair: Mr Brian Houston
Chief Executive: Tim Davison
Lothian NHS Board is the common name of Lothian Health Board

RESEARCH	SERVICE EVALUATION	CLINICAL / NON-FINANCIAL AUDIT	USUAL PRACTICE (in public health including health protection)
The attempt to derive generalisable or transferable new knowledge to answer questions with scientifically sound methods* including studies that aim to generate hypotheses as well as studies that aim to test them, in addition to simply descriptive studies.	Designed and conducted solely to define or judge current care.	Designed and conducted to produce information to inform delivery of best care.	Designed to investigate the health issues in a population in order to improve population health<br>Designed to investigate an outbreak or incident to help in disease control and prevention
Quantitative research – can be designed to test a hypothesis as in a randomised controlled trial or can simply be descriptive as in a postal survey.<br>Qualitative research – can be used to generate a hypothesis, usually identifies/explores themes.	Designed to answer: "What standard does this service achieve?"	Designed to answer: "Does this service reach a predetermined standard?"	Designed to answer: "What are the health issues in this population and how do we address them?"<br>Designed to answer: "What is the cause of this outbreak or incident and how do we manage it?"
Quantitative research - addresses clearly defined questions, aims and objectives.<br>Qualitative research – usually has clear aims and objectives but may not establish the exact questions to be asked until research is underway.	Measures current service without reference to a standard.	Measures against a standard.	Systematic, quantitative or qualitative methods may be used.
Quantitative research – may involve evaluating or comparing interventions, particularly new ones. However, some quantitative research such as descriptive surveys, do not involve interventions.<br>Qualitative research – seeks to understand better the perceptions and reasoning of people.	Involves an intervention in use only. The choice of treatment, care or services is that of the care professional and patient/service user according to guidance, professional standards and/or patient/ service user preference.	Involves an intervention in use only. The choice of treatment, care or services is that of the care professional and patient/service user according to guidance, professional standards and/or patient/service user preference.	Involves an intervention in use only. Any choice of intervention, treatment, care or services is based on best public health evidence or professional consensus.
Usually involves collecting data that are additional to those for routine care but may include data collected routinely. May involve treatments, samples or investigations additional to routine care. May involve data collected from interviews, focus groups and/or observation.	Usually involves analysis of existing data but may also include administration of interview(s) or questionnaire(s).	Usually involves analysis of existing data but may include administration of simple interview or questionnaire.	May involve analysis of existing routine data supplied under license/agreement or administration of interview or questionnaire to those in the population of interest. May also require evidence review.
Quantitative research – study design may involve allocating patients/service users/healthy volunteers to an intervention.<br>Qualitative research – does not usually involve allocating participants to an intervention.	No allocation to intervention: the care professional and patient/ service user have chosen intervention before service evaluation.	No allocation to intervention: the care professional and patient/service user have chosen intervention before audit.	No allocation to intervention.
May involve randomisation.	No randomisation.	No randomisation.	May involve randomisation but not for treatment/ care/ intervention.
Normally requires REC review but not always. Refer to http://hradecisiontools.org.uk/ethics/ for more information.	Does not require REC review.	Does not require REC review.	Does not require REC review.

Published October 2017 © Health Research Authority 2017. Copyright and other intellectual property rights in this material belong to the HRA and all rights are reserved. The HRA authorises UK healthcare organisations to reproduce this material for educational and non-commercial use.

Headquarters
Waverley Gate, 2-4 Waterloo Place
Edinburgh EH1 3EG
Chair: Mr Brian Houston
Chief Executive: Tim Davison
Lothian NHS Board is the common name of Lothian Health Board

* UK Policy Framework for Health and Social Care Research definition of research:

> *"3.1 For the purpose of this policy framework, research is defined as the attempt to derive generalisable or transferable1 new2 knowledge to answer or refine relevant questions with scientifically sound methods3. This excludes audits of practice and service evaluations. It includes activities that are carried out in preparation for or as a consequence of the interventional part4 of the research, such as screening potential participants for eligibility, obtaining participants' consent and publishing results. It also includes noninterventional health and social care research (i.e. projects that do not involve any change in standard treatment, care or other services), projects that aim to generate hypotheses, methodological research and descriptive research. Projects whose primary purpose is educational to the researcher, either in obtaining an educational qualification or in otherwise acquiring research skills, but which also fall into the definition of research, are in scope of this policy framework. Activities that are not research according to this definition should not be presented as research and need not be conducted or managed in accordance with this framework. A decision tool that provides a definitive answer about whether a project counts as research under this policy framework is available at www.hradecisiontools.org.uk/research.*

---

1 NB This definition involves an attempt at generalisability or transferability, i.e. the project deliberately uses methods intended to achieve quantitative or qualitative findings that can be applied to settings or contexts other than those in which they were tested. The actual generalisability or transferability of some research findings may only become apparent once the project has been completed.

2 Including new knowledge about existing treatments or care.

3 Projects that are not designed well enough to meet this definition are not exempt from this policy framework – see paragraph 9.10.a.

4 This means the part of the research where a change in treatment, care or other services is made for the purpose of the research. It does not refer to other methodological 'interventions', e.g. issuing a postal survey.

Published October 2017 © Health Research Authority 2017. Copyright and other intellectual property rights in this material belong to the HRA and all rights are reserved. The HRA authorises UK healthcare organisations to reproduce this material for educational and non-commercial use.

Headquarters
Waverley Gate, 2-4 Waterloo Place
Edinburgh EH1 3EG
Chair: Mr Brian Houston
Chief Executive: Tim Davison
Lothian NHS Board is the common name of Lothian Health Board
