# Supplementary material for: Quality and outcomes in global cancer surgery: protocol for a multicentre, international, prospective cohort study (GlobalSurg 3)
Source: BMJ Open. 2019 May 24;9(5):e026646. doi: 10.1136/bmjopen-2018-026646 (PMC6538014; doi:10.1136/bmjopen-2018-026646)
Supplement: Supplementary file 1 [file bmjopen-2018-026646supp001.pdf]

SUPPLEMENTARY FILE 1. Required data fields for all patients

**Patient characteristics**	
Patient ID	Local hospital field
Primary method of patient identification	Multidisciplinary team meeting / tumour board list, outpatient clinic list, theatre logbook, planned operating list, ward/handover list, staff memory
Age	Completed years
Gender	Male, Female, Unknown
Body mass index (weight (kg) / height^2 (metres))	Underweight (BMI <18.5)<br>Normal weight (BMI 18.5 to 24.9)<br>Overweight (BMI 25 to 30)<br>Obese (BMI >30)
Unintentional weight loss (≥10% over 6 months, include clothes size ref in key)	No, Yes, Unknown
Performance status	0, 1, 2, 3, 4, Unknown
ASA score	I, II, III, IV, V, Unknown
Smoking status	No-never, Stopped >6 weeks ago, Yes-current smoker, Unknown
Diabetes	No<br>Diet controlled<br>Medication (non-insulin) controlled<br>Insulin Controlled<br>Unknown
Human Immunodeficiency Virus (HIV) tested	No, Yes-NEGATIVE, Yes-POSITIVE
**Pathway**	
Presentation	Symptomatic, screening, detected incidentally, unknown
Date of first consult for cancer symptoms (may be estimated)	DD/MM/YYY
Who did the patient first consult for cancer symptoms?	Local clinic: family doctor / general practitioner<br>Local clinic: nurse<br>Local clinic: specialist doctor<br>Hospital: out-patient clinic<br>Hospital: in-patient<br>Other/non-medical/traditional healer<br>Unknown
Distance from home to hospital	< 10 km, 10-20 km, 20-50 km, 50-100 km, >100 km, Unknown
**Disease characteristics**	
Location	Breast, Gastric, Colorectal
Cancer specific information	*Fixed fields for each cancer (see specific cancer variables)*
Diagnosis (what tests were performed pre-operatively, please tick all that apply)	*Fixed fields for each cancer (see specific cancer variables)*
Clinical stage	TNM classification / Essential TNM Classification
Neoadjuvant therapy	*Fixed fields for each cancer (see specific cancer variables)*
**Operative characteristics**	
Date of admission	DD/MM/YY, 24 hour clock
Date and time of operation	DD/MM/YY, 24 hour clock
Urgency of operation	Elective, Emergency
Surgical intent (at completion of procedure)	Palliative, Curative
Was a surgical safety checklist used?	No-but available in this hospital, No-but available in this hospital, Yes, Unknown
Primary operation performed	*Fixed fields for each cancer (see specific cancer variables)*
**Pathology**	
Most valid basis for cancer diagnosis	Clinical only<br>Imaging<br>Exploratory surgery/endoscopy without histology<br>Tumour specific markers<br>Cytology<br>Histology of metastasis (secondary deposit)<br>Histology of primary
Histology	*Fixed fields for each cancer (see specific cancer variables)*
Size of invasive tumour	Centimetres

TNM (pathology)	
Number of INVOLVED lymph nodes in specimen	
TOTAL number of lymph nodes in specimen	
Histological grade	1, 2, 3, 4
Lymphatic or vascular invasion	No, Yes, Unknown
Resection margins	Fixed fields for each cancer (see specific cancer variables)
**Outcomes and adjuvant treatment**	
Length of postoperative stay	Continuous number of days
How was 30-day follow-up status achieved? (dropdown box)	Still an inpatient OR re-admitted<br>Clinic review<br>Telephone review<br>Community/home review<br>Discharged before 30 days and not contacted again
30-day mortality (if alive at the point of discharge and no follow-up information available, indicate Alive)	Alive, Dead (date of death), Unknown
30-day cancer-specific complications	Fixed fields for each cancer (see specific cancer variables)
30-day minor complication (CD I)	No, Yes, Unknown
30-day minor complication (CD II)	No, Yes, Unknown
30-day unexpected re-intervention (CD III)	No, Yes-NOT under general anaesthetic, Yes-under anaesthetic, Unknown
30-day unplanned critical care admission (CD IV)	No, Yes-single organ failure, Yes-multi organ failure, Unknown
30-day unplanned hospital readmission	No, Yes, Unknown
Surgical site infection	No<br>Yes, no treatment/wound opened only (CD I)<br>Yes, antibiotics only (CD II)<br>Yes, return to operating theatre (CD III)<br>Yes, requiring critical care admission (CD IV)<br>Yes, resulting in death (CD V)<br>Unknown
Post-operative haemorrhage	No<br>Yes, no intervention required (CD I)<br>Yes, drug treatment only (CD II)<br>Yes, intervention required (CD III)<br>Yes, critical care admission &/- intervention required (CD IV)<br>Yes, resulting in death (CD V)<br>Unknown
Planned adjuvant treatment	*Fixed fields for each cancer (see specific cancer variables)*

Those written in italics represent variables which have cancer-specific data points
