# Supplementary material for: Quality and outcomes in global cancer surgery: protocol for a multicentre, international, prospective cohort study (GlobalSurg 3)
Source: BMJ Open. 2019 May 24;9(5):e026646. doi: 10.1136/bmjopen-2018-026646 (PMC6538014; doi:10.1136/bmjopen-2018-026646)
Supplement: Supplementary file 2 [file bmjopen-2018-026646supp002.pdf]

SUPPLEMENTARY FILE 2. Required data fields for patients with breast cancer

**Disease characteristics**	
Diagnosis (what tests were performed pre-operatively, please tick all that apply)	> USS (No-not available, No-not indicated, No-indicated and facilities available, but patient not able to pay, Yes, Unknown)<br>> CT (No-not available, No-not indicated, No-indicated and facilities available, but patient not able to pay, Yes, Unknown)<br>> MRI (No-not available, No-not indicated, No-indicated and facilities available, but patient not able to pay, Yes, Unknown)<br>> Mammogram (No-not available, No-not indicated, No-indicated and facilities available, but patient not able to pay, Yes, Unknown)<br>> Fine needle aspiration (No-not available, No-not indicated, No-indicated and facilities available, but patient not able to pay, Yes, Unknown)<br>> Core biopsy (No-not available, No-not indicated, No-indicated and facilities available, but patient not able to pay, Yes, Unknown)<br>> Open/excision biopsy (No-not available, No-not indicated, No-indicated and facilities available, but patient not able to pay, Yes, Unknown)<br>> ER, PR, Ki-67, HER2 status assessed (No-not available in this hospital, No-but available in this hospital, Yes-NEGATIVE, Yes-POSITIVE, Unknown)
Stage (dropdown box)	TNM classification / Essential TNM classification<br>Unknown
Neoadjuvant chemotherapy	No, patient does not need it<br>No, patient needs it, but not available<br>No, patient needs it, facilities available, but patient not able to pay<br>No, planned but not given<br>Yes, NO anthracycline, NO taxane<br>Yes, anthracycline, NO taxane<br>Yes, anthracycline AND taxane<br>Yes, regimen unknown<br>Unknown
Neoadjuvant radiotherapy	No, patient does not need it<br>No, patient needs it, but not available<br>No, patient needs it, facilities available, but patient not able to pay<br>No, planned but not given<br>Yes (Cobalt)<br>Yes (Linear accelerator)<br>Yes (type unknown)<br>Unknown
Other neoadjuvant treatment (tick all that apply)	Hormone therapy<br>Biological therapy (HER2 inhibitor)<br>Oophrectomy<br>Other (free text)
**Operation**	
Primary operation	Mastectomy<br>Partial mastectomy / wide local excision / lumpectomy<br>Open biopsy of breast<br>Other operations on breast
Sentinel lymph node biopsy	No, not available in this hospital<br>No, but available in this hospital<br>Yes, single technique<br>Yes, dual technique<br>Unknown
Axillary lymph node biopsy	No, Yes, Unknown
Resection margins checked at time of surgery	No, not available in this hospital<br>No, but available in this hospital<br>Yes, by x-ray<br>Yes, by frozen section

	Unknown
Reconstruction	No, not available in this hospital<br>No, but available in this hospital<br>Yes, immediate – prosthesis<br>Yes, immediate – flap<br>Yes, planned at later stage
**Pathology**	
Histology	Invasive ductal carcinoma<br>Invasive lobular carcinoma<br>Ductal carcinoma in-situ (DCIS)<br>Other CANCER (specify)<br>Other BENIGN (specify)<br>Unknown, not available in this hospital<br>Unknown, but available in this hospital
Receptor status	ER, PR, Ki67, HER2<br>No-not available in this hospital, No-but available in this hospital, Yes-NEGATIVE, Yes-POSITIVE, Unknown
Resection margins	< 1 mm / tumour on inked margin<br>1-5 mm (NO tumour on inked margin)<br>>5 mm<br>Margins confirmed clear, but no distance given<br>Unknown, not available in this hospital<br>Unknown, but available in this hospital
**Outcomes and Adjuvant treatment**	
Post-operative seroma	No<br>Yes, no intervention/aspiration only (CD I)<br>Yes, antibiotic treatment only (CD II)<br>Yes, intervention required (CD III)<br>Yes, critical care admission &/- intervention (CD IV)<br>Yes, resulting in death (CD V)<br>Unknown
Planned adjuvant treatment (tick all that apply)	No, patient does not need it<br>No, patient needs it, but not available<br>No, patient needs it, facilities available, patient unable to pay<br>Yes, in this hospital<br>Yes, in another hospital in this country<br>Yes, in another hospital in a different country<br><br>Chemotherapy<br>Radiotherapy<br>Biological therapy (HER2 inhibitor)<br>Hormone therapy<br>Other (free text)
