# Supplementary material for: Quality and outcomes in global cancer surgery: protocol for a multicentre, international, prospective cohort study (GlobalSurg 3)
Source: BMJ Open. 2019 May 24;9(5):e026646. doi: 10.1136/bmjopen-2018-026646 (PMC6538014; doi:10.1136/bmjopen-2018-026646)
Supplement: Supplementary file 3 [file bmjopen-2018-026646supp003.pdf]

SUPPLEMENTARY FILE 3. Required data fields for patients with gastric cancer

**Disease characteristics**	
Diagnostic (what tests were performed pre-operatively, please tick all that apply)	> USS (No-not available, No-not indicated, No, indicated and facilities available, but patient not able to pay), Yes, Unknown)<br>> CT (No-not available, No-not indicated, No, indicated and facilities available, but patient not able to pay), Yes, Unknown)<br>> MRI (No-not available, No-not indicated, No, indicated and facilities available, but patient not able to pay), Yes, Unknown)<br>> Endoscopy (No-not available, No-not indicated, No, indicated and facilities available, but patient not able to pay), Yes, Unknown)<br>> Biopsy (No-not available, No-not indicated, No, indicated and facilities available, but patient not able to pay), Yes, Unknown)<br>> Staging laparoscopy (No-not available, No-not indicated, No, indicated and facilities available, but patient not able to pay), Yes, Unknown)
Stage (dropdown box)	TNM classification / Essential TNM classification
Neoadjuvant chemotherapy	No, patient does not need it<br>No, patient needs it, but not available<br>No, patient needs it, facilities available, but patient not able to pay<br>No, planned but not given<br>Yes<br>Unknown
Neoadjuvant radiotherapy	No, patient does not need it<br>No, patient needs it, but not available<br>No, patient needs it, facilities available, but patient not able to pay<br>No, planned but not given<br>Yes (Cobalt)<br>Yes (Linear accelerator)<br>Yes (type unknown)<br>Unknown
Other neoadjuvant treatment (tick all that apply)	Other (free text)
**Operation**	
Primary operation	Abdomen: Laparotomy with no other procedure<br>Abdomen: Diagnostic laparoscopy with no other procedure<br>Stomach: Total excision of stomach<br>Stomach: Partial excision of stomach<br>Stomach: Connection of stomach to jejunum<br>Stomach: Other open operations on stomach
Operative approach	Open<br>Laparoscopic (+/- open specimen extraction)<br>Laparoscopic converted to open<br>Robotic<br>Robotic converted to open
Site	Upper third (cardia/fundus)<br>Middle third (body)<br>Distal third (antrum/pylorus)<br>Entire stomach<br>Unknown
Cancer specific information	> Anastomosis: Not performed, handsewn, stapled, unknown<br>> D2 lymphadenectomy performed: No, Yes, Unknown<br>> Obstructed: No, Yes, Unknown<br>> Perforated: No, Yes, Unknown
**Pathology**	
Histology (dropdown box)	Adenocarcinoma<br>Lymphoma<br>Gastrointestinal stromal tumour (GIST)<br>Carcinoid<br>Other CANCER (specify)<br>Other BENIGN (specify)

	Unknown, histology not available in this hospital<br>Unknown, but histology available in this hospital
HER2 receptor status tested (on surgical resection specimen)	No-not available in this hospital, No-but available in this hospital, Yes-NEGATIVE, Yes-POSITIVE, Unknown
Resection margins	No residual disease (R0)<br>Microscopic residual disease (R1)<br>Macroscopic residual disease (R2)<br>Unknown, not available in this hospital<br>Unknown, but available in this hospital
**Outcomes and adjuvant treatment**	
Intra-abdominal abscess	No<br>Yes, no intervention (CD I)<br>Yes, antibiotics only (CD II)<br>Yes, surgical/radiological drainage (CD III)<br>Yes, critical care admission (CD IV)<br>Yes, resulting in death (CD V)<br>Unknown
Anastomotic leak	No<br>Yes, no intervention required (CD I)<br>Yes, drug treatment only (CD II)<br>Yes, intervention required (CD III)<br>Yes, critical care admission &/- intervention required (CD IV)<br>Yes, resulting in death (CD V)<br>Unknown
Planned adjuvant treatment (tick all that apply)	No, patient does not need it<br>No, patient needs it, but not available<br>No, patient needs it, facilities available, patient unable to pay<br>Yes, in this hospital<br>Yes, in another hospital in this country<br>Yes, in another hospital in a different country<br><br>Chemotherapy<br>Radiotherapy<br>Biological therapy (HER2 inhibitor)<br>Hormone therapy<br>HIPEC<br>Other (free text)
