# Supplementary material for: Quality and outcomes in global cancer surgery: protocol for a multicentre, international, prospective cohort study (GlobalSurg 3)
Source: BMJ Open. 2019 May 24;9(5):e026646. doi: 10.1136/bmjopen-2018-026646 (PMC6538014; doi:10.1136/bmjopen-2018-026646)
Supplement: Author's manuscript [file bmjopen-2018-026646.draft_revisions.pdf]

BMJ Open is committed to open peer review. As part of this commitment we make the peer review history of every article we publish publicly available.

When an article is published we post the peer reviewers' comments and the authors' responses online. We also post the versions of the paper that were used during peer review. These are the versions that the peer review comments apply to.

The versions of the paper that follow are the versions that were submitted during the peer review process. They are not the versions of record or the final published versions. They should not be cited or distributed as the published version of this manuscript.

BMJ Open is an open access journal and the full, final, typeset and author-corrected version of record of the manuscript is available on our site with no access controls, subscription charges or pay-per-view fees (http://bmjopen.bmj.com).

If you have any questions on BMJ Open's open peer review process please email info.bmjopen@bmj.com

BMJ Open

Quality and outcomes in global cancer surgery: protocol for a multicentre, international, prospective cohort study (GlobalSurg 3)

Journal:	*BMJ Open*
Manuscript ID	bmjopen-2018-026646
Article Type:	Protocol
Date Submitted by the Author:	24-Sep-2018
Complete List of Authors:	on Global Surgery, NIHR Global Health Research Unit ; University of Edinburgh, NIHR Unit on Global Surgery (Universities of Birmingham, Edinburgh and Warwick)<br>Harrison, Ewen; NIHR Unit on Global Surgery (Universities of Birmingham, Edinburgh and Warwick)
Keywords:	Cancer, Global Surgery, Perioperative Complications, Mortality, Quality, Perioperative Care

SCHOLARONE™
Manuscripts

Title page

Quality and outcomes in global cancer surgery: protocol for a multicentre, international, prospective cohort study (GlobalSurg 3)

Author:
NIHR Global Health Research Unit on Global Surgery
Collaborating authors are shown at the end of the manuscript

Correspondence:
Ewen Harrison
NIHR Unit on Global Surgery (Universities of Birmingham, Edinburgh and Warwick)
University of Edinburgh
Clinical Surgery
Royal Infirmary of Edinburgh
Edinburgh EH16 4SA

Email: enquiry@globalsurg.org / ewen.harrison@gmail.com
Telephone: 0131 242 3616
Twitter: @GlobalSurg
Web: globalsurg.org

Word count: 2572

Keywords: Cancer; Global Surgery; Perioperative Complications; Mortality; Quality; Perioperative Care

Clinical Trials identifier: NCT03471494

Funding: Funded by a Department for International Development (DFID) - Medical Research Council (MRC) - Wellcome Trust Joint Global Health Trial Development Grant (MR/N022114/1). A National Institute of Health Research (NIHR) Global Health Research Unit Grant (NIHR 17-0799) is supporting the establishment of surgical research units in a subset of contributing low-income countries. The views

For peer review only - http://bmjopen.bmj.com/site/about/guidelines.xhtml

expressed are those of the authors and not necessarily those of the NHS, the NIHR or the UK Department of Health and Social Care.

Conflicts of interest: Funding as declared. GlobalSurg is run by the Surgical Research Gateway (SuRG) Foundation. The SuRG Foundation is a registered charity (charity number 1159898) whose object is to advanced the education of medical students and doctors in surgical science, clinical research and audit methods by promoting participation in collaborative clinical research and audit studies.

For peer review only

For peer review only - http://bmjopen.bmj.com/site/about/guidelines.xhtml

ABSTRACT

Introduction

Empirical, observational data relating to the diagnosis, management and outcome of three common worldwide cancers requiring surgery is lacking. However, it has been demonstrated that patients in low- and middle-income countries undergoing surgery for cancer are at increased risk of death and major complications post-operatively. This study aims to determine quality and outcomes in breast, gastric and colorectal cancer surgery across worldwide hospital settings.

Methods and analysis

This multicentre, international prospective cohort study will be undertaken by any hospital providing emergency or elective surgical services for breast, gastric or colorectal cancer. Centres will collect observational data on consecutive patients undergoing primary emergency or elective surgery for breast, gastric or colorectal cancer during a 6-month period. The primary outcome is the incidence of mortality and major complication rate at 30-days after cancer surgery. Infrastructure and care processes in the treatment of these cancers worldwide will also be characterised.

Ethics and dissemination

This project will not affect clinical practice and has been classified as clinical audit following research ethics review. The protocol will be disseminated through the international GlobalSurg network.

Trail registration number

NCT03471494

For peer review only - http://bmjopen.bmj.com/site/about/guidelines.xhtml

Strengths and limitations of this study

- This will be the first international, multicentre, prospective study to assess quality and outcomes in patients undergoing surgery for three of the most common global cancers
- The collaborative methodology adopted by our group, as described elsewhere, has previously delivered two large high-quality studies, while avoiding overburdening low-resource centres that may otherwise be unable to participate in such projects
- Definitions of quality in surgical cancer care are disputed and little evidence exists of their validity or appropriateness in low- and middle-income countries; high quality data will help identify specific measures for cancer care in resource-limited settings
- Only those patients undergoing primary surgery for breast, gastric or colorectal cancers will be included, and therefore outcomes in patients receiving only conservative or oncological therapy will not be included
- As strict primary data monitoring is not possible within the limitations of the study, we will use a previously developed mixed-methods validation process

For peer review only

For peer review only - http://bmjopen.bmj.com/site/about/guidelines.xhtml

INTRODUCTION

Of the 15.2 million individuals diagnosed with cancer in 2015, 80% required surgery.[1] In tumours amenable to surgical resection, surgery often offers the best chance of cure, particularly in early-stage disease. It has been estimated that 45 million surgical procedures are needed each year worldwide, yet fewer than 25% of patients with cancer have access to safe, affordable, and timely surgery. While death rates from cancer are decreasing in high-income countries, the opposite has been demonstrated in low- and middle-income countries (LMICs).[2] Up to 1.5% of the gross domestic product is lost because of cancer in some LMIC regions.[3]

Our recent LMIC-led three-stage research prioritisation exercise identified cancer surgery as a major research priority. Breast cancer, gastric cancer, and colorectal cancer, represent a significant burden of disease across income settings.[1-2] Yet, most studies that examine the global distribution and outcomes of solid cancers use simulated methods due to the absence of robust data, including country-specific information on cancer epidemiology, stage distribution, and treatment approaches.[1]

Our previous prospective, observational cohort studies GlobalSurg 1 and 2[4-5] have demonstrated that patients in LMICs have an increased risk of death and complications following gastrointestinal cancer surgery. These differences persisted in multivariable models accounting for confounders in mortality (OR 3.18, 95% CI 2.12-4.76), major complication (2.14, 1.19-3.84) and SSI (1.32, 1.04-1.68) at 30 days after surgery. Post-operative complications can have a more severe consequences in LMICs, including death, long-term disability, and catastrophic healthcare expenditure.[6]

For peer review only - http://bmjopen.bmj.com/site/about/guidelines.xhtml

The measures used to determine the quality of surgical cancer care are controversial and subject to on-going debate. Guidelines produced by bodies such as the National Institute for Health and Care Excellence (NICE, UK) and American College of Surgeons in high-income countries provide some consensus.[7-8] However, there is little evidence on the appropriateness of such guidelines in LMICs or what specific measures may indicate quality in cancer surgery in resource-poor settings.

The aim of the GlobalSurg 3 Study is to determine variation in the quality of cancer surgery worldwide, focusing on patient outcomes, infrastructure and care processes. This study is driven from within our well-established global network and will be performed in upwards of 85 countries.

Primary aims

The primary aim is to audit 30-day mortality and complication rates after cancer surgery across low-, middle- and high-human development index (HDI) countries.

Secondary aims

The secondary aim is to measure the quality of surgical cancer care and is designed to be relevant in low-, middle-, and high-income settings. Conditional data points will be dependent on the specific resources available in a hospital and will include infrastructure, care process measures, and outcomes.

For peer review only - http://bmjopen.bmj.com/site/about/guidelines.xhtml

METHODS AND ANALYSIS

This is a multicentre, international, prospective, observational cohort study of all consecutive patients undergoing surgery for breast, gastric or colorectal cancer over a 28-day period. Individual collaborators are free to choose any 28-day period within the 6-month study period to collect data. This ‘snapshot’ study design is a validated model that has been delivered successfully in previous studies.[4-5,9]

The research collaborative

GlobalSurg (http://globalsurg.org/) is a collaboration between practising surgeons from around the world, performing research in surgery to foster local, national and international research networks. The collaborative model used has previously been described elsewhere[10] and has already facilitated two multicentre, international, prospective cohort studies including a total of 26 228 patients undergoing emergency and elective abdominal surgery.[4-5] The NIHR Unit on Global Surgery was established in 2017 and is a consortium between the Universities of Birmingham, Edinburgh and Warwick, together with international partners. The Units objective is to advance the education of medical students and doctors in surgical science, clinical research and audit methods by promoting participation in collaborative clinical research and audit studies.

Study setting

Any surgical unit providing emergency or elective surgery for breast, gastric or colorectal cancer worldwide is eligible to participate. An eligible hospital is not required to perform surgery for all three conditions; however, consecutive patients

For peer review only - http://bmjopen.bmj.com/site/about/guidelines.xhtml

with breast, gastric or colorectal cancer managed surgically in an individual centre must be collected during the specified study period.

Included centres must capture all consecutive patients and ensure data collection is >90% complete. Centres with >10% missing data, when including all data points, will be excluded from the final analysis and removed from the authorship. There is no minimum number of patients per centre, as long as all eligible patients treated during the study period are included. Multiple teams covering different non-overlapping time periods at each hospital are encouraged.

Patient inclusion and exclusion criteria

Adult patients aged 18 or over undergoing emergency or elective surgery for breast, gastric or colorectal cancer are eligible to enter. Any operative approach or treatment intent can be used. Patients whose primary pathology is not suspected to be breast, gastric or colorectal cancer; have a recurrence of their cancer; or are undergoing a procedure that does not require a skin incision should be excluded (Box 1). Each individual patient should only be included once into the study.

BOX 1. Patient inclusion and exclusion criteria

**Inclusion criteria**
• Adult patients aged 18 or over<br>• Consecutive patients undergoing therapeutic surgery (curative or palliative) for breast, gastric, and colorectal cancer<br>• Patients with suspected benign pathology pre-operatively whom were

For peer review only

For peer review only - http://bmjopen.bmj.com/site/about/guidelines.xhtml

subsequently found to have a diagnosis of cancer following their surgery<br>• Undergoing emergency or elective procedure requiring a skin incision performed under general or neuraxial (e.g. regional, epidural or spinal) anaesthesia.<br>• Includes open, laparoscopic, laparoscopic converted and robotic cases
**Exclusion criteria**
• Operations with a sole diagnostic or staging intent<br>• Procedures which do not require a skin incision<br>• Patients with recurrence of breast, gastric or colorectal cancer

For peer review only

Outcome Measures

The primary outcome measure is the rate of mortality and major complication within 30 days of surgery. Major complications will be defined as occurrence of a Clavien-Dindo[11] grade III or IV (Box 2) complication within 30-days of index operation, where day of operation is day 0.

BOX 2. Clavien-Dindo classification of major post-operative complications[11]

Clavien-Dindo grade III
Unplanned surgical, endoscopic or radiological intervention<br>IIIa: intervention not under general anaesthesia;<br>IIIb: intervention under general anaesthesia
Clavien-Dindo grade IV
Life-threatening complication requiring unplanned critical care management<br>IVa: single organ dysfunction (including dialysis);

For peer review only - http://bmjopen.bmj.com/site/about/guidelines.xhtml

IVb: multiorgan dysfunction

The secondary outcomes that will be derived from this study include incidence of surgical site infection and predefined cancer-specific quality measures for infrastructure and outcomes in cancer care (Box 3-5).

For peer review only

BOX 3. Breast cancer quality measures

Infrastructure and care processes
Availability and performance of:<br><br>• Pre-operative fine needle aspiration/core biopsy to diagnose breast cancer<br>• Breast/axillary MRI for staging<br>• Breast conservation surgery for AJCC stage 0/I/II breast cancer<br>• Axillary/breast radiotherapy and axillary lymph node clearance (at least 10 lymph nodes for analysis)<br>• Sentinel lymph-node biopsy for early invasive breast cancer<br>• Progesterone receptor (PR), oestrogen receptor (ER), human epidermal growth factor receptor 2 (HER2) receptor and Ki67 status for invasive cancers<br>• Treatment with adjuvant treatment where appropriate within 31 days of completion of surgery<br>• Plan for radiotherapy for all with breast conserving surgery with clear margins (including DCIS)<br>• Treatment decisions made within multidisciplinary team meeting / tumour board
Outcomes

For peer review only - http://bmjopen.bmj.com/site/about/guidelines.xhtml

- 30-day complication rate of surgical site infection, abscess formation, seroma, unplanned reoperation, unplanned readmission and requirement for unplanned critical care
- Margin involvement (or ability to measure this locally) with “tumour on inked margin” or a margin <2 mm in DCIS considered positive

For peer review only

BOX 4. Gastric cancer quality measures

Infrastructure and care processes
Availability/performance of:<br>• Endoscopy and biopsy to reach a diagnosis of cancer<br>• CT chest, abdomen and pelvis scan performed for pre-operative staging<br>• Pre- or post-operative chemotherapy for gastric cancer<br>• Treatment decisions made within multidisciplinary team meeting / tumour board
Outcomes
• 30-day complication rate of surgical site infection, anastomotic leak, unplanned reoperation, and requirement for unplanned critical care<br>• At least 15 regional lymph nodes removed and pathologically examined for resected gastric cancer (or ability to measure this locally)

BOX 5. Colorectal cancer quality measures

For peer review only - http://bmjopen.bmj.com/site/about/guidelines.xhtml

Infrastructure and care processes
Availability/performance of: • CT chest, abdomen and pelvis scan performed for pre-operative staging • Pre-operative MRI for rectal cancer • Planning and treatment with post-operative chemotherapy following resection for lymph node positive colon cancer • Treatment with pre-operative chemotherapy/radiotherapy • Treatment decisions made within multidisciplinary team meeting / tumour board • Stoma formation rate
Outcomes
• 30-day complication rate of surgical site infection, anastomotic leak, unplanned reoperation, unplanned readmission and requirement for unplanned critical care • Circumferential resection margin (CRM) >1mm (or ability to measure this locally) • At least 12 regional lymph nodes removed and pathologically examined for resected colon cancer (or ability to measure this locally)

Data points

Data points relating to patient characteristics, cancer staging, neoadjuvant therapy, operative treatment and postoperative period will be collected (Supplementary files 1-4). In order to maximise data completion, a minimal dataset has been designed including factors only relevant to quality and outcome measures in surgery for cancer. Review by international collaborators within the GlobalSurg Collaborative has also

For peer review only - http://bmjopen.bmj.com/site/about/guidelines.xhtml

ensured the dataset is relevant to cancer surgery in a worldwide setting. Investigators will enter data via the secure internet-based Research Electronic Data Capture (REDCap) system.[12] Anonymous patient data will be held on the system hosted by the University of Edinburgh, Scotland, UK.

Investigators

The study will be undertaken by investigators around the world who will be responsible for disseminating the protocol at their individual site, ensuring appropriate study approvals are in place, identifying and including all eligible patients during each four week data collection period and responsible for accurate uploading of data to an online REDCap database.

A central study writing committee comprising of an internationally representative group of healthcare professionals will be responsible for data analysis, final manuscript drafting and submission. Individuals will be required to register their unit via the REDCap system and will be required to complete a training module prior to commencing data collection.

Countries with multiple sites will be assigned a country lead, who will be responsible for coordinating multiple teams across sites to ensure duplication of data does not occur. Where individual hospitals have a large number of local coordinators, a hospital lead will be appointed to aid coordination. A maximum of three local investigators can cover each 4-week data collection period, with the collection of multiple, non-overlapping collection periods by the same or different local

For peer review only - http://bmjopen.bmj.com/site/about/guidelines.xhtml

investigators in a single centre possible. They will be responsible for gaining local audit, service evaluation or research ethics approval as appropriate to their institution.

Investigators will create clear mechanisms appropriate to their institution to identify and include all eligible patients, involving daily review of operating logbooks, multidisciplinary team meeting, admission and handover lists. This will include identifying clear pathways to accurately collect baseline, cancer-specific and follow-up data within the normal limits of follow-up. Local arrangements may include daily review of the patient and notes focussed on included data points, reviewing patient status in outpatient clinics or via telephone interview at 30-days (if this is normal practice) and checking for re-admission through handover lists. All investigators will be listed as collaborators on resulting publications in accordance with previous consensus guidelines for collaborative group research.[13]

Quality of data

To ensure high data quality, a detailed protocol has been produced and published online. Translations into 12 common languages has also been performed to ease investigator understanding, including Arabic, French, Hindi, Italian, Mandarin, Portuguese, Russian, Spanish and Swahili. Collaborators are encouraged to perform data input in real-time using the REDCap system, with an individual patient record requiring to be completed before submission is possible. Data quality rules will also ensure data quality, highlighting disparities in data fields to the local collaborator for review. Online training is available to collaborators prior to the commencement of data collection at their institution, detailing secure REDCap data entry, patient outcome assessment and disease-specific parameters.

For peer review only - http://bmjopen.bmj.com/site/about/guidelines.xhtml

Data validation

Data validation will be performed in two parts across a group of representative centres similar to the structure successfully used in previous studies of this nature.[5] Case ascertainment assessment will involve an independent investigator determining the number of eligible cases within a 4-week data collection centre and comparing this to the actual number of cases submitted. By comparing samples, a quantitative estimate of case ascertainment will be produced by the central data team. Secondly, validators will be asked to provide data for a subset of variables, two patient variables, two operation variables and two outcome measures in order to measure data accuracy.

Statistical analysis and power calculation

Variation across different international health settings will be tested using the HDI[14], a composite statistic of life expectancy, education, and income indices published by the United Nations. Bayesian multilevel logistic regression models will be constructed to account for case mix, with population stratification by hospital and country of residence incorporated as random effects with constrained gradients. Further pre-specified subgroup analyses will be made by geographical country grouping, cancer-type, emergency vs. elective surgery, performance status, palliative vs. curative surgery, extent of staging, and extent of pathological analyses. Data will not be analysed or reported at an individual surgeon or hospital level. Following analysis, results will be fed back to participants at the centre level, but no other centres will be identifiable.

For peer review only - http://bmjopen.bmj.com/site/about/guidelines.xhtml

Estimates of 30-day mortality for gastrointestinal cancer resection were determined using data from the GlobalSurg 1 and 2 studies[4-5]. Stratification of results by human development index was performed, with prominent variation in 30-day mortality rate between high HDI and low/middle HDI groups seen after cancer surgery in both emergency surgery (11.6% (75/644) vs. 27.3% (59/216)) and elective surgery (2.0% (30/1501) vs. 5.5% (23/416)). An indicative sample size calculation using the smaller of these estimates suggests around 500 patients per group at 80% power (p1=0.020, p2=0.055, alpha=0.05) or 640 patients per group at 90% power would be required to conclude a difference in 30-day mortality rate between HDI groups.

Patient and public involvement

Patient representatives for GlobalSurg, from both the United Kingdom and Rwanda, guided development of the research question, outcomes measured and study design. Patients were not involved in the recruitment or conduct of the study. We aim to publish the study results as open access, which will be readily available to patients and the public.

Trial registration number

This study has been registered with ClinicalTrials.gov (identifier: NCT03471494). The registration is available to view at: https://clinicaltrials.gov/ct2/show/NCT03471494.

For peer review only - http://bmjopen.bmj.com/site/about/guidelines.xhtml

ETHICS AND DISSEMINATION

Research ethics approval

The primary audit standards stems from the UK National Institute for Health Clinical Excellence[7] and the American College of Surgeons Commission of Cancer Quality of Care[8] guidelines for the diagnosis, investigation and management of breast, gastric and colorectal cancer (Box 6). As this study will not change local clinical practice and is limited to using data obtained as part of usual care, it has been classified as an audit by the South Scotland Research Ethics Service in Edinburgh, Scotland (Supplementary file 5). Therefore this may be considered a global audit or global service evaluation. Local investigators will be responsible for ensuring the study is registered appropriately and approval gained from the relevant local clinical audit departments, research and development department or institutional review boards. If such departments are unavailable, written permission should be supplied by the chief of surgery or responsible supervising consultant/attending physician.

BOX 6. Study audit standards

Breast cancer
• American College of Surgeons Commission of Cancer Quality of Care for Breast Cancer[8]<br>• National Institute for Health and Care Excellence (NICE): Early and locally advanced breast cancer: diagnosis and treatment; Clinical Guideline CG80[17]<br>• SSO/ASTRO consensus guidelines for early stage breast cancer[18]
Gastric cancer
• American College of Surgeons Commission of Cancer Quality of Care for Gastric

For peer review only - http://bmjopen.bmj.com/site/about/guidelines.xhtml

For peer review only

Cancer[8]<br>• National Institute for Health and Care Excellence (NICE): Oesophago-gastric cancer: assessment and management in adults[19]
Colorectal cancer
• American College of Surgeons Commission of Cancer Quality of Care for Colorectal Cancer[8]<br>• National Institute for Health and Care Excellence (NICE): Colorectal cancer: diagnosis and management; Clinical Guideline CG131[20]

Protocol dissemination

The protocol will be disseminated across the established GlobalSurg network, compromised of surgeons, medical students and clinical staff across the world. The network previously included over 1800 collaborators across 343 centres representing 66 countries.[5] Country leads are responsible for local co-ordination and dissemination within their country. In addition, the use of social media including Facebook, Twitter and YouTube has been shown to be an effective medium for dissemination of such collaborative projects[15] and will also be employed.

Dissemination of results

We aim to publish the study results as open access. Data from the study will be described to ensure individual countries, hospitals and surgeons are anonymous and then shall be deposited in an online data repository for others to analyse. On completion of the study, participating centres will be provided with their own benchmark performance and access to interactive web-based applications to use for

For peer review only - http://bmjopen.bmj.com/site/about/guidelines.xhtml

quality improvement or subsequent re-audit. Based on the results of the GlobalSurg 3 study, feasibility studies investigating the collection of other outcome measures relating to cancer surgery and development of quality improvement and/or interventional clinical trials will be suggested for possible implementation in surgical cancer units for each included hospital in the study.

For peer review only

DISCUSSION

In this study protocol, we describe a multicentre, international, prospective cohort study investigating the quality and outcomes of surgery for three of the most common global cancers. Despite the likely increased risk of mortality and major morbidity for patients undergoing surgery for cancer in low- and middle-income countries, high quality, empirical data is currently unavailable. Furthermore, in countries with limited resources applicability of cancer surgery guidelines are yet to be tested.

By using a collaborative methodology and a short 4-week data collection period, the study will recruit sufficient patients to measure this, while avoiding burdening low-resource centres that may otherwise be unable to participate. Investigating the morbidity and mortality caused by cancer surgery globally, this study will provide a platform to build future quality improvement programmes and interventional trials as previously demonstrated by the GlobalSurg network.

This study will be delivered using an international multidisciplinary collaborative network of healthcare researchers, with the collaborative model having consistently proven its ability to produce high-quality outcomes in international studies.[4-5] A detailed study protocol in multiple languages, mandatory training, data quality control and validation period will ensure standardisation to deliver a reliable and accurate data set.

As the second most common cause of death in 2015, with 8.7 million deaths globally[2], cancer incidence is predicted to become an increasing burden worldwide[1-2] and place further pressure on already limited healthcare systems.

For peer review only - http://bmjopen.bmj.com/site/about/guidelines.xhtml

Neoplasms already contribute to significant global morbidity and mortality, causing the highest loss of gross domestic product of any surgical disease.[3] Surgery can provide cure for many cancers, particularly in countries where limited access to oncology treatment exists. However, the majority of the world's population lack access to safe, affordable and timely cancer surgery.[16]

This study provides the first opportunity to collect and analyse prospective, observational data for three of the most common global cancers. Current literature is heavily reliant on simulated models based on limited data sources.[2-3,16] Our study will quantify any global inequalities in cancer surgery, highlight differences in patient presentation, treatment interventions and surgical outcomes.

With feedback of outcomes and specific quality measures relating to each cancer, collaborators will have the opportunity to appraise their current practice against a global standard. Furthermore, surgeons and other interested parties will be able to use the findings from this study to help develop focussed cancer surgery guidelines based on empirical global data.

Finally, this study will continue to strengthen the international GlobalSurg network, further developing capacity for research in LMICs. Focussed interventional trials derived from study findings will follow, aimed at improving global outcomes in cancer surgery.

For peer review only

For peer review only - http://bmjopen.bmj.com/site/about/guidelines.xhtml

ACKNOWLEDGEMENTS

We would like to thank Emmy Runigamugabo and Azmina Verjee for their advice and contribution as patient representatives in the development of this study protocol.

For peer review only

For peer review only - http://bmjopen.bmj.com/site/about/guidelines.xhtml

REFERENCES

1. Sullivan R, Alatise OI, Anderson BO et al. Global cancer surgery: delivering safe, affordable, and timely cancer surgery. *Lancet Oncol* 2015;16(11):1193–1224.

2. Global Burden of Disease Cancer Collaboration. Global, Regional, and National Cancer Incidence, Mortality, Years of Life Lost, Years Lived With Disability, and Disability-Adjusted Life-years for 32 Cancer Groups, 1990 to 2015: A Systematic Analysis for the Global Burden of Disease Study. *JAMA Oncol* 2017;3(4):524.

3. Alkire BC, Raykar NP, Shrime MG,et al. Global access to surgical care: a modelling study. *Lancet Glob Health* 2015;1;3(6):e316–23.

4. GlobalSurg Collaborative. Mortality of emergency abdominal surgery in high-, middle- and low-income countries. British Journal of Surgery. 2016;103(8):971-88.

5. GlobalSurg Collaborative. Surgical site infection after gastrointestinal surgery in high-income, middle-income, and low-income countries: a prospective, international, multicentre cohort study. *Lancet Infect Dis* 2018;3099(18):1-10.

6. Shrime MG, Dare A, Alkire BC, et al. A global country-level comparison of the financial burden of surgery. *Br J Surg* 2016 Oct;103(11):1453-61.

7. National Institute for Clinical Excellence. NICE Guidance [cited 2018 August 3]. https://www.nice.org.uk

For peer review only

8. American College of Surgeons. Commission on Cancer Quality of Care Measures [cited 2018 June 27]. https://www.facs.org/quality-programs/cancer/ncdb/qualitymeasures

9. Pinkney TD, Calvert M, Bartlett DC, et al. Impact of wound edge protection devices on surgical site infection after laparotomy: multicentre randomised controlled trial (ROSSINI Trial). *BMJ* 2013;347:f4305.

10. Bhangu A, Kolias AG, Pinkney T, et al. Surgical research collaboratives in the UK. *Lancet* 2013 Sep 28;382(9898):1091-2.

11. Dindo D, Demartines N, Clavien P-A. Classification of Surgical Complications: A New Proposal With Evaluation in a Cohort of 6336 Patients and Results of a Survey. *Ann Surg* 2004;240(2):205-213.

12. Harris PA, Taylor R, Thielke R, et al. Research electronic data capture (REDCap) - a metadata-driven methodology and workflow process for providing translational research informatics support. *J Biomed Inform* 2009;42:377–81.

13. National Research Council. Recognising contributions to work in research collaboratives: Guidelines for standardising reporting of authorship in collaborative research. *Int J Surg* 2018;52:355.

14. United Nations Development Programme (UNDP). Human Development Data (1990-2015) [cited 2018 June 27]. hdr.undp.org/en/statistics

For peer review only - http://bmjopen.bmj.com/site/about/guidelines.xhtml

For peer review only

15. Khatri C, Chapman SJ, Glasbey J, *et al.* Social media and internet driven study recruitment: evaluating a new model for promoting collaborator engagement and participation. *PLoS One* 2015;10:e0118899.

16. Alkire BC, Shrime MG, Dare AJ, et al. The Global Economic Consequences of Selected Surgical Diseases: A Modelling Study. *Lancet Glob Health*. 2015;3(Suppl 2):S21-S27.

17. National Institute for Clinical Excellence Guidance and guidelines. Early and locally advanced breast cancer: Diagnosis and treatment [cited 2018 June 27]. https://www.nice.org.uk/guidance/cg80

18. Moran MS, Schnitt SJ, Giuliano AE, et al. SSO-ASTRO Consensus Guideline on Margins for Breast-Conserving Surgery with Whole Breast Irradiation in Stage I and II Invasive Breast Cancer. *Int J Radiat Oncol Biol Phys* 2014;88(3):553–64.

19. National Institute for Clinical Excellence Guidance and guidelines. Oesophago-gastric cancer: assessment and management in adults [cited 2018 June 27]. https://www.nice.org.uk/guidance/NG83

20. National Institute for Clinical Excellence Guidance and guidelines. Colorectal cancer: diagnosis and management [cited 2018 June 27]. https://www.nice.org.uk/guidance/cg131

For peer review only - http://bmjopen.bmj.com/site/about/guidelines.xhtml

AUTHORS CONTRIBUTIONS

All authors contributed to the design, drafting and review of this study protocol.

FULL AUTHORSHIP LIST

NIHR Global Health Research Unit on Global Surgery

Protocol development group: Stephen R Knight, Thomas M Drake, Dmitri Nepogodiev, J Edward F Fitzgerald, Adesoji O Ademuyiwa, Philip Alexander, Jean C Allen Ingabire, Sara W. Al-Saqqa, Bruce Biccard, Giuliano Borda, David Borowski, Sule Burger, Kathryn Chu, Damian Clarke, Ainhoa Costa, Justine Davies, Rachel Donaldson, Chikwendu Ede, O. James Garden, Dhruv Ghosh, James C. Glasbey, T Peter Kingham, Hosni Khairy Salem, Anyomih Theophilus Kojo, Zach Koto, Marie Carmela Lapitan, Ismail Lawani, Chiapo Lesetedi, Maria Lorena Aguilera, Charles Mabedi, Mayaba Maimbo, Laura Magill, Felix Makinde Alakaloko, Alex Makupe, Janet Martin, Antonio Ramos-De la Medina, Mark Monahan, Rachel Moore, Vanessa Msosa, Soloman Mulira, Alphonse Zeta Mutabazi, Elmi Muller, Joseph Musowoyo, Adewale Oluseye Adisa, Jean Léon Olory-Togbe, Riinu Ots, Ahmad Uzair Qureshi, Sarah Rayne, Tracey Roberts, Marie Dione Parreno-Sacdalan, Catherine Shaw, Neil Smart, Martin Smith, Richard Spence, Steph van Straten, Stephen Tabiri, Viki Tayler, Thomas G Weiser, John Windsor, Joseph Yorke, Raul Yepez, Richard Lilford, Dion Morton, Aneel Bhangu, Sudha Sundar, Ewen M. Harrison

For peer review only

For peer review only - http://bmjopen.bmj.com/site/about/guidelines.xhtml

Funding statement

This study was funded by Department for International Development (DFID) – Medical Research Council (MRC) - Wellcome Trust Joint Global Health Trial Development Grant (MR/N022114/1). A National Institute of Health Research (NIHR) Global Health Research Unit Grant (NIHR 17-0799) is supporting the establishment of surgical research units in a subset of contributing low-income countries. The views expressed are those of the authors and not necessarily those of the NHS, the NIHR or the UK Department of Health and Social Care.

Competing interests

None declared. GlobalSurg is run by the Surgical Research Gateway (SuRG) Foundation. The SuRG Foundation is a registered charity (charity number 1159898) whose object is to advanced the education of medical students and doctors in surgical science, clinical research and audit methods by promoting participation in collaborative clinical research and audit studies.

Patient consent

This study was reviewed by the South East Scotland Research Ethics committee and they have classified this as clinical audit/service evaluation in the UK. As such, this does not require specific consent to be obtained from patients.

Ethics approval

South East Scotland Research Ethics Service in Edinburgh

Word count: 2572

For peer review only - http://bmjopen.bmj.com/site/about/guidelines.xhtml

SUPPLEMENTARY FILE 1. Required data fields for all patients

**Patient characteristics**	
Patient ID	Local hospital field
Primary method of patient identification	Multidisciplinary team meeting / tumour board list, outpatient clinic list, theatre logbook, planned operating list, ward/handover list, staff memory
Age	Completed years
Gender	Male, Female, Unknown
Body mass index (weight (kg) / height^2 (metres))	Underweight (BMI <18.5)<br>Normal weight (BMI 18.5 to 24.9)<br>Overweight (BMI 25 to 30)<br>Obese (BMI >30)
Unintentional weight loss (≥10% over 6 months, include clothes size ref in key)	No, Yes, Unknown
Performance status	0, 1, 2, 3, 4, Unknown
ASA score	I, II, III, IV, V, Unknown
Smoking status	No-never, Stopped >6 weeks ago, Yes-current smoker, Unknown
Diabetes	No<br>Diet controlled<br>Medication (non-insulin) controlled<br>Insulin Controlled<br>Unknown
Human Immunodeficiency Virus (HIV) tested	No, Yes-NEGATIVE, Yes-POSITIVE
**Pathway**	
Presentation	Symptomatic, screening, detected incidentally, unknown
Date of first consult for cancer symptoms (may be estimated)	DD/MM/YYY
Who did the patient first consult for cancer symptoms?	Local clinic: family doctor / general practitioner<br>Local clinic: nurse<br>Local clinic: specialist doctor<br>Hospital: out-patient clinic<br>Hospital: in-patient<br>Other/non-medical/traditional healer<br>Unknown
Distance from home to hospital	< 10 km, 10-20 km, 20-50 km, 50-100 km, >100 km, Unknown
**Disease characteristics**	
Location	Breast, Gastric, Colorectal
Cancer specific information	*Fixed fields for each cancer (see specific cancer variables)*
Diagnosis (what tests were performed pre-operatively, please tick all that apply)	*Fixed fields for each cancer (see specific cancer variables)*
Clinical stage	TNM classification / Essential TNM Classification
Neoadjuvant therapy	*Fixed fields for each cancer (see specific cancer variables)*
**Operative characteristics**	
Date of admission	DD/MM/YY, 24 hour clock
Date and time of operation	DD/MM/YY, 24 hour clock
Urgency of operation	Elective, Emergency
Surgical intent (at completion of procedure)	Palliative, Curative
Was a surgical safety checklist used?	No-but available in this hospital, No-but available in this hospital, Yes, Unknown
Primary operation performed	*Fixed fields for each cancer (see specific cancer variables)*
**Pathology**	
Most valid basis for cancer diagnosis	Clinical only<br>Imaging<br>Exploratory surgery/endoscopy without histology<br>Tumour specific markers<br>Cytology<br>Histology of metastasis (secondary deposit)<br>Histology of primary
Histology	*Fixed fields for each cancer (see specific cancer variables)*
Size of invasive tumour	Centimetres

For peer review only

TNM (pathology)	
Number of INVOLVED lymph nodes in specimen	
TOTAL number of lymph nodes in specimen	
Histological grade	1, 2, 3, 4
Lymphatic or vascular invasion	No, Yes, Unknown
Resection margins	Fixed fields for each cancer (see specific cancer variables)
**Outcomes and adjuvant treatment**	
Length of postoperative stay	Continuous number of days
How was 30-day follow-up status achieved? (dropdown box)	Still an inpatient OR re-admitted<br>Clinic review<br>Telephone review<br>Community/home review<br>Discharged before 30 days and not contacted again
30-day mortality (if alive at the point of discharge and no follow-up information available, indicate Alive)	Alive, Dead (date of death), Unknown
30-day cancer-specific complications	Fixed fields for each cancer (see specific cancer variables)
30-day minor complication (CD I)	No, Yes, Unknown
30-day minor complication (CD II)	No, Yes, Unknown
30-day unexpected re-intervention (CD III)	No, Yes-NOT under general anaesthetic, Yes-under anaesthetic, Unknown
30-day unplanned critical care admission (CD IV)	No, Yes-single organ failure, Yes-multi organ failure, Unknown
30-day unplanned hospital readmission	No, Yes, Unknown
Surgical site infection	No<br>Yes, no treatment/wound opened only (CD I)<br>Yes, antibiotics only (CD II)<br>Yes, return to operating theatre (CD III)<br>Yes, requiring critical care admission (CD IV)<br>Yes, resulting in death (CD V)<br>Unknown
Post-operative haemorrhage	No<br>Yes, no intervention required (CD I)<br>Yes, drug treatment only (CD II)<br>Yes, intervention required (CD III)<br>Yes, critical care admission &/- intervention required (CD IV)<br>Yes, resulting in death (CD V)<br>Unknown
Planned adjuvant treatment	*Fixed fields for each cancer (see specific cancer variables)*

Those written in italics represent variables which have cancer-specific data points

For peer review only

SUPPLEMENTARY FILE 2. Required data fields for patients with breast cancer

**Disease characteristics**	
Diagnosis (what tests were performed pre-operatively, please tick all that apply)	> USS (No-not available, No-not indicated, No-indicated and facilities available, but patient not able to pay, Yes, Unknown)<br>> CT (No-not available, No-not indicated, No-indicated and facilities available, but patient not able to pay, Yes, Unknown)<br>> MRI (No-not available, No-not indicated, No-indicated and facilities available, but patient not able to pay, Yes, Unknown)<br>> Mammogram (No-not available, No-not indicated, No-indicated and facilities available, but patient not able to pay, Yes, Unknown)<br>> Fine needle aspiration (No-not available, No-not indicated, No-indicated and facilities available, but patient not able to pay, Yes, Unknown)<br>> Core biopsy (No-not available, No-not indicated, No-indicated and facilities available, but patient not able to pay, Yes, Unknown)<br>> Open/excision biopsy (No-not available, No-not indicated, No-indicated and facilities available, but patient not able to pay, Yes, Unknown)<br>> ER, PR, Ki-67, HER2 status assessed (No-not available in this hospital, No-but available in this hospital, Yes-NEGATIVE, Yes-POSITIVE, Unknown)
Stage (dropdown box)	TNM classification / Essential TNM classification<br>Unknown
Neoadjuvant chemotherapy	No, patient does not need it<br>No, patient needs it, but not available<br>No, patient needs it, facilities available, but patient not able to pay<br>No, planned but not given<br>Yes, NO anthracycline, NO taxane<br>Yes, anthracycline, NO taxane<br>Yes, anthracycline AND taxane<br>Yes, regimen unknown<br>Unknown
Neoadjuvant radiotherapy	No, patient does not need it<br>No, patient needs it, but not available<br>No, patient needs it, facilities available, but patient not able to pay<br>No, planned but not given<br>Yes (Cobalt)<br>Yes (Linear accelerator)<br>Yes (type unknown)<br>Unknown
Other neoadjuvant treatment (tick all that apply)	Hormone therapy<br>Biological therapy (HER2 inhibitor)<br>Oophrectomy<br>Other (free text)
**Operation**	
Primary operation	Mastectomy<br>Partial mastectomy / wide local excision / lumpectomy<br>Open biopsy of breast<br>Other operations on breast
Sentinel lymph node biopsy	No, not available in this hospital<br>No, but available in this hospital<br>Yes, single technique<br>Yes, dual technique<br>Unknown
Axillary lymph node biopsy	No, Yes, Unknown
Resection margins checked at time of surgery	No, not available in this hospital<br>No, but available in this hospital<br>Yes, by x-ray<br>Yes, by frozen section

For peer review only

	Unknown
Reconstruction	No, not available in this hospital<br>No, but available in this hospital<br>Yes, immediate – prosthesis<br>Yes, immediate – flap<br>Yes, planned at later stage
**Pathology**	
Histology	Invasive ductal carcinoma<br>Invasive lobular carcinoma<br>Ductal carcinoma in-situ (DCIS)<br>Other CANCER (specify)<br>Other BENIGN (specify)<br>Unknown, not available in this hospital<br>Unknown, but available in this hospital
Receptor status	ER, PR, Ki67, HER2<br>No-not available in this hospital, No-but available in this hospital, Yes-NEGATIVE, Yes-POSITIVE, Unknown
Resection margins	< 1 mm / tumour on inked margin<br>1-5 mm (NO tumour on inked margin)<br>>5 mm<br>Margins confirmed clear, but no distance given<br>Unknown, not available in this hospital<br>Unknown, but available in this hospital
**Outcomes and Adjuvant treatment**	
Post-operative seroma	No<br>Yes, no intervention/aspiration only (CD I)<br>Yes, antibiotic treatment only (CD II)<br>Yes, intervention required (CD III)<br>Yes, critical care admission &/- intervention (CD IV)<br>Yes, resulting in death (CD V)<br>Unknown
Planned adjuvant treatment (tick all that apply)	No, patient does not need it<br>No, patient needs it, but not available<br>No, patient needs it, facilities available, patient unable to pay<br>Yes, in this hospital<br>Yes, in another hospital in this country<br>Yes, in another hospital in a different country<br><br>Chemotherapy<br>Radiotherapy<br>Biological therapy (HER2 inhibitor)<br>Hormone therapy<br>Other (free text)

For peer review only

SUPPLEMENTARY FILE 3. Required data fields for patients with gastric cancer

**Disease characteristics**	
Diagnostic (what tests were performed pre-operatively, please tick all that apply)	> USS (No-not available, No-not indicated, No, indicated and facilities available, but patient not able to pay), Yes, Unknown)<br>> CT (No-not available, No-not indicated, No, indicated and facilities available, but patient not able to pay), Yes, Unknown)<br>> MRI (No-not available, No-not indicated, No, indicated and facilities available, but patient not able to pay), Yes, Unknown)<br>> Endoscopy (No-not available, No-not indicated, No, indicated and facilities available, but patient not able to pay), Yes, Unknown)<br>> Biopsy (No-not available, No-not indicated, No, indicated and facilities available, but patient not able to pay), Yes, Unknown)<br>> Staging laparoscopy (No-not available, No-not indicated, No, indicated and facilities available, but patient not able to pay), Yes, Unknown)
Stage (dropdown box)	TNM classification / Essential TNM classification
Neoadjuvant chemotherapy	No, patient does not need it<br>No, patient needs it, but not available<br>No, patient needs it, facilities available, but patient not able to pay<br>No, planned but not given<br>Yes<br>Unknown
Neoadjuvant radiotherapy	No, patient does not need it<br>No, patient needs it, but not available<br>No, patient needs it, facilities available, but patient not able to pay<br>No, planned but not given<br>Yes (Cobalt)<br>Yes (Linear accelerator)<br>Yes (type unknown)<br>Unknown
Other neoadjuvant treatment (tick all that apply)	Other (free text)
**Operation**	
Primary operation	Abdomen: Laparotomy with no other procedure<br>Abdomen: Diagnostic laparoscopy with no other procedure<br>Stomach: Total excision of stomach<br>Stomach: Partial excision of stomach<br>Stomach: Connection of stomach to jejunum<br>Stomach: Other open operations on stomach
Site	Upper third (cardia/fundus)<br>Middle third (body)<br>Distal third (antrum/pylorus)<br>Entire stomach<br>Unknown
Cancer specific information	> Anastomosis: Not performed, handsewn, stapled, unknown<br>> D2 lymphadenectomy performed: No, Yes, Unknown<br>> Obstructed: No, Yes, Unknown<br>> Perforated: No, Yes, Unknown
**Pathology**	
Histology (dropdown box)	Adenocarcinoma<br>Lymphoma<br>Gastrointestinal stromal tumour (GIST)<br>Carcinoid<br>Other CANCER (specify)<br>Other BENIGN (specify)<br>Unknown, histology not available in this hospital<br>Unknown, but histology available in this hospital

For peer review only

HER2 receptor status tested (on surgical resection specimen)	No-not available in this hospital, No-but available in this hospital, Yes-NEGATIVE, Yes-POSITIVE, Unknown
Resection margins	No residual disease (R0)<br>Microscopic residual disease (R1)<br>Macroscopic residual disease (R2)<br>Unknown, not available in this hospital<br>Unknown, but available in this hospital
**Outcomes and adjuvant treatment**	
Intra-abdominal abscess	No<br>Yes, no intervention (CD I)<br>Yes, antibiotics only (CD II)<br>Yes, surgical/radiological drainage (CD III)<br>Yes, critical care admission (CD IV)<br>Yes, resulting in death (CD V)<br>Unknown
Anastomotic leak	No<br>Yes, no intervention required (CD I)<br>Yes, drug treatment only (CD II)<br>Yes, intervention required (CD III)<br>Yes, critical care admission &/- intervention required (CD IV)<br>Yes, resulting in death (CD V)<br>Unknown
Planned adjuvant treatment (tick all that apply)	No, patient does not need it<br>No, patient needs it, but not available<br>No, patient needs it, facilities available, patient unable to pay<br>Yes, in this hospital<br>Yes, in another hospital in this country<br>Yes, in another hospital in a different country<br><br>Chemotherapy<br>Radiotherapy<br>Biological therapy (HER2 inhibitor)<br>Hormone therapy<br>HIPEC<br>Other (free text)

For peer review only

For peer review only

SUPPLEMENTARY FILE 4: Required data fields for patients with colorectal cancer

**Disease characteristics**	
Diagnostic (what tests were performed pre-operatively, please tick all that apply)	> USS (No-not available, No-not indicated, No-indicated and facilities available, but patient not able to pay, Yes, Unknown)<br>> CT (No-not available, No-not indicated, No-indicated and facilities available, but patient not able to pay, Yes, Unknown)<br>> MRI (No-not available, No-not indicated, No-indicated and facilities available, but patient not able to pay, Yes, Unknown)<br>> Endoscopy (No-not available, No-not indicated, No-indicated and facilities available, but patient not able to pay, Yes, Unknown)<br>> Biopsy (No-not available, No-not indicated, No-indicated and facilities available, but patient not able to pay, Yes, Unknown)<br>> Staging laparoscopy (No-not available, No-not indicated, No-indicated and facilities available, but patient not able to pay, Yes, Unknown)
Stage	TNM classification / Essential TNM classification<br>Unknown
Neoadjuvant chemotherapy	No, patient does not need it<br>No, patient needs it, but not available<br>No, patient needs it, facilities available, but patient not able to pay<br>No, planned but not given<br>Yes<br>Unknown
Neoadjuvant radiotherapy	No, patient does not need it<br>No, patient needs it, but not available<br>No, patient needs it, facilities available, but patient not able to pay<br>No, planned but not given<br>Yes (Cobalt)<br>Yes (Linear accelerator)<br>Yes (type unknown)<br>Unknown
Other neoadjuvant treatment (tick all that apply)	Other (free text)
**Operation**	
Primary operation	Abdomen: Laparotomy with no other procedure<br>Abdomen: Diagnostic laparoscopy with no other procedure<br>Small bowel: Formation of ileostomy only<br>Colon: Total excision of colon and rectum<br>Colon: Total excision of colon<br>Colon: Extended excision of right hemicolon<br>Colon: Excision of right hemicolon<br>Colon: Excision of transverse colon<br>Colon: Excision of left hemicolon<br>Colon: Excision of sigmoid colon<br>Colon: Other excision of colon<br>Colon: Formation of any colonic stoma<br>Colon: Other open operations on colon<br>Rectum: Abdominoperineal resection<br>Rectum: Resection with anastomosis of colon to anus<br>Rectum: Anterior resection with anastomosis<br>Rectum: Resection with closure of rectal stump (Hartmann's)<br>Rectum: Other open operations on rectum
Cancer specific information	> Site: Caecum, Ascending colon, Transverse colon, Descending colon, Sigmoid colon, High rectum (>10 to 15cm from anal verge), Middle Rectum (>5 to 10cm), Low rectum (≤5cm), Unknown<br>> Anastomosis: Not performed, handsewn, stapled, unknown<br>> Obstructed: No, Yes, Unknown<br>> Perforated: No, Yes, Unknown
Stoma formation	No,

For peer review only

	Yes, loop ileostomy<br>Yes, end ileostomy<br>Yes, loop colostomy<br>Yes, end colostomy<br>Unknown
**Pathology**	
Histology (dropdown box)	Adenocarcinoma<br>Squamous cell carcinoma<br>Carcinoid<br>Lymphoma<br>Other CANCER (specify)<br>Other BENIGN (specify)<br>Unknown, histology not available in this hospital<br>Unknown, but histology available in this hospital
Perineural invasion	No, Yes, Unknown
Resection margins	No residual disease (R0)<br>Microscopic residual disease (R1)<br>Macroscopic residual disease (R2)<br>Unknown, not available in this hospital<br>Unknown, but available in this hospital
Circumferential margin (CRM)	Millimetres
**Outcomes and adjuvant treatment**	
Anastomotic leak	No<br>Yes, no intervention required<br>Yes, intervention required<br>Yes, critical care admission +/- intervention required<br>Unknown
Planned adjuvant treatment (tick all that apply)	No, patient does not need it<br>No, patient needs it, but not available<br>No, patient needs it, facilities available, patient unable to pay<br>Yes, in this hospital<br>Yes, in another hospital in this country<br>Yes, in another hospital in a different country<br><br>Chemotherapy<br>Radiotherapy<br>Biological therapy (HER2 inhibitor)<br>Hormone therapy<br>Liver resection (metastasis)<br>Lung resection (metastasis)<br>HIPEC<br>Other (free text)

For peer review only

South East Scotland Research Ethics Service

Waverley Gate
2-4 Waterloo Place
Edinburgh
EH1 3EG

NHS Lothian

Ewen M Harrison

Senior Lecturer in General Surgery /
Honorary Consultant Surgeon
NIHR Unit on Global Surgery (Universities of Birmingham, Edinburgh and Warwick)
Clinical Surgery
University of Edinburgh
Royal Infirmary of Edinburgh
Edinburgh
EH16 4SA

Date: 19/02/2018
Your Ref:
Our Ref: NR/161AB6
Enquiries to:
Direct Line: 0131 465 5679
Email:

Dear Mr Harrison,

Project Title: "GlobalSurg 3: Quality and outcomes in global cancer surgery: a prospective, international cohort study"

You have sought advice from the South East Scotland Research Ethics Service on the above project. This has been considered by the Scientific Officer and you are advised that, based on the email correspondence it does not need NHS ethical review under the terms of the Governance Arrangements for Research Ethics Committees (A Harmonised Edition).

If the project is considered to be health-related research you will require a sponsor and ethical approval as outlined in The Research Governance Framework for Health and Community Care. You may wish to contact your employer or professional body to arrange this. You may also require NHS management permission (R&D approval). You should contact the relevant NHS R&D departments to organise this.

For projects that are not research and will be conducted within the NHS you should contact the relevant local clinical governance team who will inform you of the relevant governance procedures required before the project commences.

This letter should not be interpreted as giving a form of ethical approval or any endorsement of the project, but it may be provided to a journal or other body as evidence that NHS ethical approval is not required. However, if you, your sponsor/funder feel that the project requires ethical review by an NHS REC, please write setting out your reasons and we will be pleased to consider further. You should retain a copy of this letter with your project file as evidence that you have sought advice from the South East Scotland Research Ethics Service.

Yours sincerely,

Helen Newbery
Scientific Officer
South East Scotland Research Ethics Service

For peer review only

Headquarters
Waverley Gate, 2-4 Waterloo Place
Edinburgh EH1 3EG
Chair: Mr Brian Houston
Chief Executive: Tim Davison
Lothian NHS Board is the common name of Lothian Health Board

For peer review only - http://bmjopen.bmj.com/site/about/guidelines.xhtml

South East Scotland Research Ethics Service

RESEARCH	SERVICE EVALUATION	CLINICAL / NON-FINANCIAL AUDIT	USUAL PRACTICE (in public health including health protection)
The attempt to derive generalisable or transferable new knowledge to answer questions with scientifically sound methods* including studies that aim to generate hypotheses as well as studies that aim to test them, in addition to simply descriptive studies.	Designed and conducted solely to define or judge current care.	Designed and conducted to produce information to inform delivery of best care.	Designed to investigate the health issues in a population in order to improve population health<br>Designed to investigate an outbreak or incident to help in disease control and prevention
Quantitative research – can be designed to test a hypothesis as in a randomised controlled trial or can simply be descriptive as in a postal survey.<br>Qualitative research – can be used to generate a hypothesis, usually identifies/explores themes.	Designed to answer: "What standard does this service achieve?"	Designed to answer: "Does this service reach a predetermined standard?"	Designed to answer: "What are the health issues in this population and how do we address them?"<br>Designed to answer: "What is the cause of this outbreak or incident and how do we manage it?"
Quantitative research - addresses clearly defined questions, aims and objectives.<br>Qualitative research – usually has clear aims and objectives but may not establish the exact questions to be asked until research is underway.	Measures current service without reference to a standard.	Measures against a standard.	Systematic, quantitative or qualitative methods may be used.
Quantitative research – may involve evaluating or comparing interventions, particularly new ones. However, some quantitative research such as descriptive surveys, do not involve interventions.<br>Qualitative research – seeks to understand better the perceptions and reasoning of people.	Involves an intervention in use only. The choice of treatment, care or services is that of the care professional and patient/service user according to guidance, professional standards and/or patient/ service user preference.	Involves an intervention in use only. The choice of treatment, care or services is that of the care professional and patient/service user according to guidance, professional standards and/or patient/service user preference.	Involves an intervention in use only. Any choice of intervention, treatment, care or services is based on best public health evidence or professional consensus.
Usually involves collecting data that are additional to those for routine care but may include data collected routinely. May involve treatments, samples or investigations additional to routine care. May involve data collected from interviews, focus groups and/or observation.	Usually involves analysis of existing data but may also include administration of interview(s) or questionnaire(s).	Usually involves analysis of existing data but may include administration of simple interview or questionnaire.	May involve analysis of existing routine data supplied under license/agreement or administration of interview or questionnaire to those in the population of interest. May also require evidence review.
Quantitative research – study design may involve allocating patients/service users/healthy volunteers to an intervention.<br>Qualitative research – does not usually involve allocating participants to an intervention.	No allocation to intervention: the care professional and patient/ service user have chosen intervention before service evaluation.	No allocation to intervention: the care professional and patient/service user have chosen intervention before audit.	No allocation to intervention.
May involve randomisation.	No randomisation.	No randomisation.	May involve randomisation but not for treatment/ care/ intervention.
Normally requires REC review but not always. Refer to http://hradecisiontools.org.uk/ethics/ for more information.	Does not require REC review.	Does not require REC review.	Does not require REC review.

Published October 2017 © Health Research Authority 2017. Copyright and other intellectual property rights in this material belong to the HRA and all rights are reserved. The HRA authorises UK healthcare organisations to reproduce this material for educational and non-commercial use.

Headquarters
Waverley Gate, 2-4 Waterloo Place
Edinburgh EH1 3EG
Chair: Mr Brian Houston
Chief Executive: Tim Davison
Lothian NHS Board is the common name of Lothian Health Board

For peer review only - http://bmjopen.bmj.com/site/about/guidelines.xhtml

South East Scotland Research Ethics Service

* UK Policy Framework for Health and Social Care Research definition of research:

> *"3.1 For the purpose of this policy framework, research is defined as the attempt to derive generalisable or transferable1 new2 knowledge to answer or refine relevant questions with scientifically sound methods3. This excludes audits of practice and service evaluations. It includes activities that are carried out in preparation for or as a consequence of the interventional part4 of the research, such as screening potential participants for eligibility, obtaining participants' consent and publishing results. It also includes noninterventional health and social care research (i.e. projects that do not involve any change in standard treatment, care or other services), projects that aim to generate hypotheses, methodological research and descriptive research. Projects whose primary purpose is educational to the researcher, either in obtaining an educational qualification or in otherwise acquiring research skills, but which also fall into the definition of research, are in scope of this policy framework. Activities that are not research according to this definition should not be presented as research and need not be conducted or managed in accordance with this framework. A decision tool that provides a definitive answer about whether a project counts as research under this policy framework is available at www.hradecisiontools.org.uk/research.*

---

1 NB This definition involves an attempt at generalisability or transferability, i.e. the project deliberately uses methods intended to achieve quantitative or qualitative findings that can be applied to settings or contexts other than those in which they were tested. The actual generalisability or transferability of some research findings may only become apparent once the project has been completed.

2 Including new knowledge about existing treatments or care.

3 Projects that are not designed well enough to meet this definition are not exempt from this policy framework – see paragraph 9.10.a.

4 This means the part of the research where a change in treatment, care or other services is made for the purpose of the research. It does not refer to other methodological 'interventions', e.g. issuing a postal survey.

Published October 2017 © Health Research Authority 2017. Copyright and other intellectual property rights in this material belong to the HRA and all rights are reserved. The HRA authorises UK healthcare organisations to reproduce this material for educational and non-commercial use.

Headquarters
Waverley Gate, 2-4 Waterloo Place
Edinburgh EH1 3EG
Chair: Mr Brian Houston
Chief Executive: Tim Davison
Lothian NHS Board is the common name of Lothian Health Board

For peer review only - http://bmjopen.bmj.com/site/about/guidelines.xhtml

STROBE 2007 (v4) Statement—Checklist of items that should be included in reports of *cohort studies*

Section/Topic	Item #	Recommendation	Reported on page #
**Title and abstract**	1	(*a*) Indicate the study's design with a commonly used term in the title or the abstract	1
		(*b*) Provide in the abstract an informative and balanced summary of what was done and what was found	3
**Introduction**			
Background/rationale	2	Explain the scientific background and rationale for the investigation being reported	5
Objectives	3	State specific objectives, including any prespecified hypotheses	6
**Methods**			
Study design	4	Present key elements of study design early in the paper	7-16
Setting	5	Describe the setting, locations, and relevant dates, including periods of recruitment, exposure, follow-up, and data collection	7-16
Participants	6	(*a*) Give the eligibility criteria, and the sources and methods of selection of participants. Describe methods of follow-up	8-16
		(*b*) For matched studies, give matching criteria and number of exposed and unexposed	Not applicable
Variables	7	Clearly define all outcomes, exposures, predictors, potential confounders, and effect modifiers. Give diagnostic criteria, if applicable	8-16
Data sources/ measurement	8*	For each variable of interest, give sources of data and details of methods of assessment (measurement). Describe comparability of assessment methods if there is more than one group	8-16
Bias	9	Describe any efforts to address potential sources of bias	14-16
Study size	10	Explain how the study size was arrived at	15
Quantitative variables	11	Explain how quantitative variables were handled in the analyses. If applicable, describe which groupings were chosen and why	14-16
Statistical methods	12	(*a*) Describe all statistical methods, including those used to control for confounding	14-16
		(*b*) Describe any methods used to examine subgroups and interactions	14-16
		(*c*) Explain how missing data were addressed	8
		(*d*) If applicable, explain how loss to follow-up was addressed	8, 13
		(*e*) Describe any sensitivity analyses	Not applicable
**Results**			

For peer review only

Participants	13*	(a) Report numbers of individuals at each stage of study—eg numbers potentially eligible, examined for eligibility, confirmed eligible, included in the study, completing follow-up, and analysed	Not applicable
		(b) Give reasons for non-participation at each stage	Not applicable
		(c) Consider use of a flow diagram	Not applicable
Descriptive data	14*	(a) Give characteristics of study participants (eg demographic, clinical, social) and information on exposures and potential confounders	Not applicable
		(b) Indicate number of participants with missing data for each variable of interest	Not applicable
		(c) Summarise follow-up time (eg, average and total amount)	Not applicable
Outcome data	15*	Report numbers of outcome events or summary measures over time	Not applicable
Main results	16	(*a*) Give unadjusted estimates and, if applicable, confounder-adjusted estimates and their precision (eg, 95% confidence interval). Make clear which confounders were adjusted for and why they were included	Not applicable
		(*b*) Report category boundaries when continuous variables were categorized	Not applicable
		(*c*) If relevant, consider translating estimates of relative risk into absolute risk for a meaningful time period	Not applicable
Other analyses	17	Report other analyses done—eg analyses of subgroups and interactions, and sensitivity analyses	Not applicable
**Discussion**			
Key results	18	Summarise key results with reference to study objectives	Not applicable
**Limitations**			
Interpretation	20	Give a cautious overall interpretation of results considering objectives, limitations, multiplicity of analyses, results from similar studies, and other relevant evidence	Not applicable
Generalisability	21	Discuss the generalisability (external validity) of the study results	Not applicable
**Other information**			
Funding	22	Give the source of funding and the role of the funders for the present study and, if applicable, for the original study on which the present article is based	1

*Give information separately for cases and controls in case-control studies and, if applicable, for exposed and unexposed groups in cohort and cross-sectional studies.

Note: An Explanation and Elaboration article discusses each checklist item and gives methodological background and published examples of transparent reporting. The STROBE checklist is best used in conjunction with this article (freely available on the Web sites of PLoS Medicine at http://www.plosmedicine.org/, Annals of Internal Medicine at http://www.annals.org/, and Epidemiology at http://www.epidem.com/). Information on the STROBE Initiative is available at www.strobe-statement.org.

For peer review only

BMJ Open

Quality and outcomes in global cancer surgery: protocol for a multicentre, international, prospective cohort study (GlobalSurg 3)

Journal:	*BMJ Open*
Manuscript ID	bmjopen-2018-026646.R1
Article Type:	Protocol
Date Submitted by the Author:	28-Dec-2018
Complete List of Authors:	on Global Surgery, NIHR Global Health Research Unit ; University of Edinburgh, NIHR Unit on Global Surgery (Universities of Birmingham, Edinburgh and Warwick)<br>Harrison, Ewen; NIHR Unit on Global Surgery (Universities of Birmingham, Edinburgh and Warwick)
**Primary Subject Heading**:	Surgery
Secondary Subject Heading:	Surgery, Global health, Oncology
Keywords:	Cancer, Global Surgery, Perioperative Complications, Mortality, Quality, Perioperative Care

SCHOLARONE™
Manuscripts

Title page

Quality and outcomes in global cancer surgery: protocol for a multicentre, international, prospective cohort study (GlobalSurg 3)

Author:
NIHR Global Health Research Unit on Global Surgery
Collaborating authors are shown at the end of the manuscript

Correspondence:
Ewen Harrison
NIHR Unit on Global Surgery (Universities of Birmingham, Edinburgh and Warwick)
University of Edinburgh
Clinical Surgery
Royal Infirmary of Edinburgh
Edinburgh EH16 4SA

Email: enquiry@globalsurg.org / ewen.harrison@gmail.com
Telephone: 0131 242 3616
Twitter: @GlobalSurg
Web: globalsurg.org

Word count: 2711

Keywords: Cancer; Global Surgery; Perioperative Complications; Mortality; Quality; Perioperative Care

Clinical Trials identifier: NCT03471494

For peer review only

ABSTRACT

Introduction

Empirical, observational data relating to the diagnosis, management and outcome of three common worldwide cancers requiring surgery is lacking. However, it has been demonstrated that patients in low- and middle-income countries undergoing surgery for cancer are at increased risk of death and major complications post-operatively. This study aims to determine quality and outcomes in breast, gastric and colorectal cancer surgery across worldwide hospital settings.

Methods and analysis

This multicentre, international prospective cohort study will be undertaken by any hospital providing emergency or elective surgical services for breast, gastric or colorectal cancer. Centres will collect observational data on consecutive patients undergoing primary emergency or elective surgery for breast, gastric or colorectal cancer during a 6-month period. The primary outcome is the incidence of mortality and major complication rate at 30-days after cancer surgery. Infrastructure and care processes in the treatment of these cancers worldwide will also be characterised.

Ethics and dissemination

This project will not affect clinical practice and has been classified as clinical audit following research ethics review. The protocol will be disseminated through the international GlobalSurg network.

Trial registration number

NCT03471494

For peer review only - http://bmjopen.bmj.com/site/about/guidelines.xhtml

Strengths and limitations of this study

- This will be the first international, multicentre, prospective study to assess quality and outcomes in patients undergoing surgery for three of the most common global cancers
- The collaborative methodology adopted by our group, as described elsewhere, has previously delivered two large high-quality studies, while avoiding overburdening low-resource centres that may otherwise be unable to participate in such projects
- Definitions of quality in surgical cancer care are disputed and little evidence exists of their validity or appropriateness in low- and middle-income countries; high quality data will help identify specific measures for cancer care in resource-limited settings
- Only those patients undergoing primary surgery for breast, gastric or colorectal cancers will be included, and therefore outcomes in patients receiving only conservative or oncological therapy will not be included
- As strict primary data monitoring is not possible within the limitations of the study, we will use a previously developed mixed-methods validation process

For peer review only

For peer review only - http://bmjopen.bmj.com/site/about/guidelines.xhtml

INTRODUCTION

Of the 15.2 million individuals diagnosed with cancer in 2015, 80% required surgery.[1] In tumours amenable to surgical resection, surgery often offers the best chance of cure, particularly in early-stage disease. It has been estimated that 45 million surgical procedures are needed each year worldwide, yet fewer than 25% of patients with cancer have access to safe, affordable, and timely surgery. While death rates from cancer are decreasing in high-income countries, the opposite has been demonstrated in low- and middle-income countries (LMICs).[2] Up to 1.5% of the gross domestic product is lost because of cancer in some LMIC regions.[3]

Our recent LMIC-led three-stage research prioritisation exercise identified cancer surgery as a major research priority. Breast cancer, gastric cancer, and colorectal cancer, represent a significant burden of disease across income settings.[1-2] Yet, most studies that examine the global distribution and outcomes of solid cancers use simulated methods due to the absence of robust data, including country-specific information on cancer epidemiology, stage distribution, and treatment approaches.[1]

Our previous prospective, observational cohort studies GlobalSurg 1 and 2[4-5] have demonstrated that patients in LMICs have an increased risk of death and complications following gastrointestinal cancer surgery. These differences persisted in multivariable models accounting for confounders in mortality (OR 3.18, 95% CI 2.12-4.76), major complication (2.14, 1.19-3.84) and SSI (1.32, 1.04-1.68) at 30 days after surgery. Post-operative complications can have a more severe consequences in LMICs, including death, long-term disability, and catastrophic healthcare expenditure.[6]

For peer review only

The measures used to determine the quality of surgical cancer care are controversial and subject to on-going debate. Guidelines produced by bodies such as the National Institute for Health and Care Excellence (NICE, UK) and American College of Surgeons in high-income countries provide some consensus.[7-8] However, there is little evidence on the appropriateness of such guidelines in LMICs or what specific measures may indicate quality in cancer surgery in resource-poor settings.

The aim of the GlobalSurg 3 Study is to determine variation in the quality of cancer surgery worldwide, focusing on patient outcomes, infrastructure and care processes. This study is driven from within our well-established global network and will be performed in upwards of 85 countries.

Primary aims

The primary aim is to audit 30-day mortality and complication rates after cancer surgery across low-, middle- and high-human development index (HDI) countries.

Secondary aims

The secondary aim is to measure the quality of surgical cancer care and is designed to be relevant in low-, middle-, and high-income settings. Conditional data points will be dependent on the specific resources available in a hospital and will include infrastructure, care process measures, and outcomes.

For peer review only - http://bmjopen.bmj.com/site/about/guidelines.xhtml

METHODS AND ANALYSIS

This is a multicentre, international, prospective, observational cohort study of all consecutive patients undergoing surgery for breast, gastric or colorectal cancer over a 28-day period. Individual collaborators are free to choose any 28-day period within the 6-month study period to collect data. This 'snapshot' study design is a validated model that has been delivered successfully in previous studies.[4-5,9]

The research collaborative

GlobalSurg (http://globalsurg.org/) is a collaboration between practising surgeons from around the world, performing research in surgery to foster local, national and international research networks. The collaborative model used has previously been described elsewhere[10] and has already facilitated two multicentre, international, prospective cohort studies including a total of 26 228 patients undergoing emergency and elective abdominal surgery.[4-5] The NIHR Unit on Global Surgery was established in 2017 and is a consortium between the Universities of Birmingham, Edinburgh and Warwick, together with international partners. The Units objective is to advance the education of medical students and doctors in surgical science, clinical research and audit methods by promoting participation in collaborative clinical research and audit studies.

Study setting

Any surgical unit providing emergency or elective surgery for breast, gastric or colorectal cancer worldwide is eligible to participate. An eligible hospital is not required to perform surgery for all three conditions; however, consecutive patients

For peer review only - http://bmjopen.bmj.com/site/about/guidelines.xhtml

with breast, gastric or colorectal cancer managed surgically in an individual centre must be collected during the specified study period.

Included centres must capture all consecutive patients and ensure data collection is >90% complete. Centres with >10% missing data, when including all data points, will be excluded from the final analysis and removed from the authorship. There is no minimum number of patients per centre, as long as all eligible patients treated during the study period are included. Multiple teams covering different non-overlapping time periods at each hospital are encouraged.

Patient inclusion and exclusion criteria

Adult patients aged 18 or over undergoing emergency or elective surgery for breast, gastric or colorectal cancer are eligible to enter. Any operative approach or treatment intent can be used. Patients whose primary pathology is not suspected to be breast, gastric or colorectal cancer; have a recurrence of their cancer; or are undergoing a procedure that does not require a skin incision should be excluded (Box 1). Each individual patient should only be included once into the study.

BOX 1. Patient inclusion and exclusion criteria

**Inclusion criteria**
• Adult patients aged 18 or over<br>• Consecutive patients undergoing therapeutic surgery (curative or palliative) for breast, gastric, and colorectal cancer

For peer review only - http://bmjopen.bmj.com/site/about/guidelines.xhtml

• Patients with suspected benign pathology pre-operatively whom were subsequently found to have a diagnosis of cancer following their surgery<br>• Undergoing emergency or elective procedure requiring a skin incision performed under general or neuraxial (e.g. regional, epidural or spinal) anaesthesia.<br>• Includes open, laparoscopic, laparoscopic converted and robotic cases
**Exclusion criteria**
• Operations with a sole diagnostic or staging intent<br>• Procedures which do not require a skin incision<br>• Patients with recurrence of breast, gastric or colorectal cancer

Outcome Measures

The primary outcome measure is the rate of mortality and major complication within 30 days of surgery. Major complications will be defined as occurrence of a Clavien-Dindo[11] grade III or IV (Box 2) complication within 30-days of index operation, where day of operation is day 0.

BOX 2. Clavien-Dindo classification of major post-operative complications[11]

Clavien-Dindo grade III
Unplanned surgical, endoscopic or radiological intervention<br>IIIa: intervention not under general anaesthesia;<br>IIIb: intervention under general anaesthesia
Clavien-Dindo grade IV
Life-threatening complication requiring unplanned critical care management

For peer review only - http://bmjopen.bmj.com/site/about/guidelines.xhtml

For peer review only

IVa: single organ dysfunction (including dialysis);

IVb: multiorgan dysfunction

The secondary outcomes that will be derived from this study include incidence of surgical site infection and predefined cancer-specific quality measures for infrastructure and outcomes in cancer care (Box 3-5).

For peer review only

BOX 3. Breast cancer quality measures

Infrastructure and care processes

Availability and performance of:

- Pre-operative fine needle aspiration/core biopsy to diagnose breast cancer
- Breast/axillary MRI for staging
- Breast conservation surgery for AJCC stage 0/I/II breast cancer
- Axillary/breast radiotherapy and axillary lymph node clearance (at least 10 lymph nodes for analysis)
- Sentinel lymph-node biopsy for early invasive breast cancer
- Progesterone receptor (PR), oestrogen receptor (ER), human epidermal growth factor receptor 2 (HER2) receptor and Ki67 status for invasive cancers
- Treatment with adjuvant treatment where appropriate within 31 days of completion of surgery
- Plan for radiotherapy for all with breast conserving surgery with clear margins (including DCIS)
- Treatment decisions made within multidisciplinary team meeting / tumour board

For peer review only - http://bmjopen.bmj.com/site/about/guidelines.xhtml

Outcomes
• 30-day complication rate of surgical site infection, abscess formation, seroma, unplanned reoperation, unplanned readmission and requirement for unplanned critical care<br>• Margin involvement (or ability to measure this locally) with “tumour on inked margin” or a margin <2 mm in DCIS considered positive

BOX 4. Gastric cancer quality measures

Infrastructure and care processes
Availability/performance of:<br>• Endoscopy and biopsy to reach a diagnosis of cancer<br>• CT chest, abdomen and pelvis scan performed for pre-operative staging<br>• Pre- or post-operative chemotherapy for gastric cancer<br>• Treatment decisions made within multidisciplinary team meeting / tumour board
Outcomes
• 30-day complication rate of surgical site infection, anastomotic leak, unplanned reoperation, and requirement for unplanned critical care<br>• At least 15 regional lymph nodes removed and pathologically examined for resected gastric cancer (or ability to measure this locally)

For peer review only - http://bmjopen.bmj.com/site/about/guidelines.xhtml

BOX 5. Colorectal cancer quality measures

Infrastructure and care processes
Availability/performance of:<br><br>• CT chest, abdomen and pelvis scan performed for pre-operative staging<br>• Pre-operative MRI for rectal cancer<br>• Planning and treatment with post-operative chemotherapy following resection for lymph node positive colon cancer<br>• Treatment with pre-operative chemotherapy/radiotherapy<br>• Treatment decisions made within multidisciplinary team meeting / tumour board<br>• Stoma formation rate
Outcomes
• 30-day complication rate of surgical site infection, anastomotic leak, unplanned reoperation, unplanned readmission and requirement for unplanned critical care<br>• Circumferential resection margin (CRM) >1mm (or ability to measure this locally)<br>• At least 12 regional lymph nodes removed and pathologically examined for resected colon cancer (or ability to measure this locally)

Data points

Data points relating to patient characteristics, cancer staging, neoadjuvant therapy, operative treatment and postoperative period will be collected (Supplementary files 1-4). In order to maximise data completion, a minimal dataset has been designed including factors only relevant to quality and outcome measures in surgery for cancer.

For peer review only - http://bmjopen.bmj.com/site/about/guidelines.xhtml

Review by international collaborators within the GlobalSurg Collaborative has also ensured the dataset is relevant to cancer surgery in a worldwide setting. Investigators will enter data via the secure internet-based Research Electronic Data Capture (REDCap) system.[12] Anonymous patient data will be held on the system hosted by the University of Edinburgh, Scotland, UK.

Investigators

The study will be undertaken by investigators around the world who will be responsible for disseminating the protocol at their individual site, ensuring appropriate study approvals are in place, identifying and including all eligible patients during each four week data collection period and responsible for accurate uploading of data to an online REDCap database.

A central study writing committee comprising of an internationally representative group of healthcare professionals will be responsible for data analysis, final manuscript drafting and submission. Individuals will be required to register their unit via the REDCap system and will be required to complete a training module prior to commencing data collection.

Countries with multiple sites will be assigned a country lead, who will be responsible for coordinating multiple teams across sites to ensure duplication of data does not occur. Where individual hospitals have a large number of local coordinators, a hospital lead will be appointed to aid coordination. A maximum of three local investigators can cover each 4-week data collection period, with the collection of multiple, non-overlapping collection periods by the same or different local

For peer review only - http://bmjopen.bmj.com/site/about/guidelines.xhtml

investigators in a single centre possible. They will be responsible for gaining local audit, service evaluation or research ethics approval as appropriate to their institution.

Investigators will create clear mechanisms appropriate to their institution to identify and include all eligible patients, involving daily review of operating logbooks, multidisciplinary team meeting, admission and handover lists. This will include identifying clear pathways to accurately collect baseline, cancer-specific and follow-up data within the normal limits of follow-up. Local arrangements may include daily review of the patient and notes focussed on included data points, reviewing patient status in outpatient clinics or via telephone interview at 30-days (if this is normal practice) and checking for re-admission through handover lists. All investigators will be listed as collaborators on resulting publications in accordance with previous consensus guidelines for collaborative group research.[13]

Quality of data

To ensure high data quality, a detailed protocol has been produced and published online. Translations into 12 common languages has also been performed to ease investigator understanding, including Arabic, French, Hindi, Italian, Mandarin, Portuguese, Russian, Spanish and Swahili. Collaborators are encouraged to perform data input in real-time using the REDCap system, with an individual patient record requiring to be completed before submission is possible. Data quality rules will also ensure data quality, highlighting disparities in data fields to the local collaborator for review. Online training is available to collaborators prior to the commencement of data collection at their institution, detailing secure REDCap data entry, patient outcome assessment and disease-specific parameters.

For peer review only - http://bmjopen.bmj.com/site/about/guidelines.xhtml

Data validation

Data validation will be performed in two parts across a group of representative centres similar to the structure successfully used in previous studies of this nature.[5] Case ascertainment assessment will involve an independent investigator determining the number of eligible cases within a 4-week data collection centre and comparing this to the actual number of cases submitted. By comparing samples, a quantitative estimate of case ascertainment will be produced by the central data team. Secondly, validators will be asked to provide data for a subset of variables, two patient variables, two operation variables and two outcome measures in order to measure data accuracy.

Statistical analysis and power calculation

Variation across different international health settings will be tested using the HDI[14], a composite statistic of life expectancy, education, and income indices published by the United Nations. Bayesian multilevel logistic regression models will be constructed to account for case mix, with population stratification by hospital and country of residence incorporated as random effects with constrained gradients.

Further pre-specified subgroup analyses will be made by geographical country grouping, cancer-type (including the separation of colonic and rectal tumours), emergency vs. elective surgery, performance status, palliative vs. curative surgery, extent of staging, and extent of pathological analyses. When assessing quality measures and processes similar patient groups will be compared, with potential confounding factors such as cancer-type, patient presentation, surgical intent and availability of adjuvant therapy accounted for within statistical models. Quality

For peer review only - http://bmjopen.bmj.com/site/about/guidelines.xhtml

metrics as described earlier in the protocol will guide exploratory analysis into the global variation in surgical management and available resources. However, it is acknowledged that such guidelines, in the majority, are designed for high-income settings and therefore their attainment will not be considered mandatory or a potential definitive measure of care quality in global cancer surgery.

Data will not be analysed or reported at an individual surgeon or hospital level. Following analysis, results will be fed back to participants at the centre level, but no other centres will be identifiable.

Estimates of 30-day mortality for gastrointestinal cancer resection were determined using data from the GlobalSurg 1 and 2 studies[4-5]. Stratification of results by human development index was performed, with prominent variation in 30-day mortality rate between high HDI and low/middle HDI groups seen after cancer surgery in both emergency surgery (11.6% (75/644) vs. 27.3% (59/216)) and elective surgery (2.0% (30/1501) vs. 5.5% (23/416)). An indicative sample size calculation using the smaller of these estimates suggests around 500 patients per group at 80% power (p1=0.020, p2=0.055, alpha=0.05) or 640 patients per group at 90% power would be required to conclude a difference in 30-day mortality rate between HDI groups.

Patient and public involvement

Patient representatives for GlobalSurg, from both the United Kingdom and Rwanda, guided development of the research question, outcomes measured and study design. Patients were not involved in the recruitment or conduct of the study. We aim to

For peer review only - http://bmjopen.bmj.com/site/about/guidelines.xhtml

publish the study results as open access, which will be readily available to patients and the public.

Trial registration number

This study has been registered with ClinicalTrials.gov (identifier: NCT03471494). The registration is available to view at: https://clinicaltrials.gov/ct2/show/NCT03471494.

For peer review only

For peer review only - http://bmjopen.bmj.com/site/about/guidelines.xhtml

ETHICS AND DISSEMINATION

Research ethics approval

The primary audit standards stems from the UK National Institute for Health Clinical Excellence[7] and the American College of Surgeons Commission of Cancer Quality of Care[8] guidelines for the diagnosis, investigation and management of breast, gastric and colorectal cancer (Box 6). As this study will not change local clinical practice and is limited to using data obtained as part of usual care, it has been classified as an audit by the South Scotland Research Ethics Service in Edinburgh, Scotland (Supplementary file 5). Therefore this may be considered a global audit or global service evaluation. Local investigators will be responsible for ensuring the study is registered appropriately and approval gained from the relevant local clinical audit departments, research and development department or institutional review boards. If such departments are unavailable, written permission should be supplied by the chief of surgery or responsible supervising consultant/attending physician.

BOX 6. Study audit standards

Breast cancer
• American College of Surgeons Commission of Cancer Quality of Care for Breast Cancer[8]<br>• National Institute for Health and Care Excellence (NICE): Early and locally advanced breast cancer: diagnosis and treatment; Clinical Guideline CG80[15]<br>• SSO/ASTRO consensus guidelines for early stage breast cancer[16]
Gastric cancer

For peer review only

• American College of Surgeons Commission of Cancer Quality of Care for Gastric Cancer[8]<br>• National Institute for Health and Care Excellence (NICE): Oesophago-gastric cancer: assessment and management in adults[17]
Colorectal cancer
• American College of Surgeons Commission of Cancer Quality of Care for Colorectal Cancer[8]<br>• National Institute for Health and Care Excellence (NICE): Colorectal cancer: diagnosis and management; Clinical Guideline CG131[18]

Protocol dissemination

The protocol will be disseminated across the established GlobalSurg network, compromised of surgeons, medical students and clinical staff across the world. The network previously included over 1800 collaborators across 343 centres representing 66 countries.[5] Country leads are responsible for local co-ordination and dissemination within their country. In addition, the use of social media including Facebook, Twitter and YouTube has been shown to be an effective medium for dissemination of such collaborative projects[19] and will also be employed.

Dissemination of results

We aim to publish the study results as open access. Data from the study will be described to ensure individual countries, hospitals and surgeons are anonymous and then shall be deposited in an online data repository for others to analyse. On completion of the study, participating centres will be provided with their own

For peer review only - http://bmjopen.bmj.com/site/about/guidelines.xhtml

benchmark performance and access to interactive web-based applications to use for quality improvement or subsequent re-audit. Based on the results of the GlobalSurg 3 study, feasibility studies investigating the collection of other outcome measures relating to cancer surgery and development of quality improvement and/or interventional clinical trials will be suggested for possible implementation in surgical cancer units for each included hospital in the study.

For peer review only

For peer review only - http://bmjopen.bmj.com/site/about/guidelines.xhtml

DISCUSSION

In this study protocol, we describe a multicentre, international, prospective cohort study investigating the quality and outcomes of surgery for three of the most common global cancers. Despite the likely increased risk of mortality and major morbidity for patients undergoing surgery for cancer in low- and middle-income countries, high quality, empirical data is currently unavailable. Furthermore, in countries with limited resources applicability of cancer surgery guidelines are yet to be tested.

By using a collaborative methodology and a short 4-week data collection period, the study will recruit sufficient patients to measure this, while avoiding burdening low-resource centres that may otherwise be unable to participate. Investigating the morbidity and mortality caused by cancer surgery globally, this study will provide a platform to build future quality improvement programmes and interventional trials as previously demonstrated by the GlobalSurg network.

This study will be delivered using an international multidisciplinary collaborative network of healthcare researchers, with the collaborative model having consistently proven its ability to produce high-quality outcomes in international studies.[4-5] A detailed study protocol in multiple languages, mandatory training, data quality control and validation period will ensure standardisation to deliver a reliable and accurate data set.

As the second most common cause of death in 2015, with 8.7 million deaths globally[2], cancer incidence is predicted to become an increasing burden worldwide[1-2] and place further pressure on already limited healthcare systems.

For peer review only - http://bmjopen.bmj.com/site/about/guidelines.xhtml

Neoplasms already contribute to significant global morbidity and mortality, causing the highest loss of gross domestic product of any surgical disease.[3] Surgery can provide cure for many cancers, particularly in countries where limited access to oncology treatment exists. However, the majority of the world's population lack access to safe, affordable and timely cancer surgery.[20]

This study provides the first opportunity to collect and analyse prospective, observational data for three of the most common global cancers. Current literature is heavily reliant on simulated models based on limited data sources.[2-3,20] Our study will quantify any global inequalities in cancer surgery, highlight differences in patient presentation, treatment interventions and surgical outcomes.

With feedback of outcomes and specific quality measures relating to each cancer, collaborators will have the opportunity to appraise their current practice against a global standard. Furthermore, surgeons and other interested parties will be able to use the findings from this study to help develop focussed cancer surgery guidelines based on empirical global data.

Finally, this study will continue to strengthen the international GlobalSurg network, further developing capacity for research in LMICs. Focussed interventional trials derived from study findings will follow, aimed at improving global outcomes in cancer surgery.

For peer review only

ACKNOWLEDGEMENTS

We would like to thank Emmy Runigamugabo and Azmina Verjee for their advice and contribution as patient representatives in the development of this study protocol.

For peer review only

For peer review only - http://bmjopen.bmj.com/site/about/guidelines.xhtml

REFERENCES

1. Sullivan R, Alatise OI, Anderson BO et al. Global cancer surgery: delivering safe, affordable, and timely cancer surgery. *Lancet Oncol* 2015;16(11):1193–1224.

2. Global Burden of Disease Cancer Collaboration. Global, Regional, and National Cancer Incidence, Mortality, Years of Life Lost, Years Lived With Disability, and Disability-Adjusted Life-years for 32 Cancer Groups, 1990 to 2015: A Systematic Analysis for the Global Burden of Disease Study. *JAMA Oncol* 2017;3(4):524.

3. Alkire BC, Raykar NP, Shrime MG,et al. Global access to surgical care: a modelling study. *Lancet Glob Health* 2015;1;3(6):e316–23.

4. GlobalSurg Collaborative. Mortality of emergency abdominal surgery in high-, middle- and low-income countries. British Journal of Surgery. 2016;103(8):971-88.

5. GlobalSurg Collaborative. Surgical site infection after gastrointestinal surgery in high-income, middle-income, and low-income countries: a prospective, international, multicentre cohort study. *Lancet Infect Dis* 2018;3099(18):1-10.

6. Shrime MG, Dare A, Alkire BC, et al. A global country-level comparison of the financial burden of surgery. *Br J Surg* 2016 Oct;103(11):1453-61.

7. National Institute for Clinical Excellence. NICE Guidance [cited 2018 August 3]. https://www.nice.org.uk

For peer review only - http://bmjopen.bmj.com/site/about/guidelines.xhtml

8. American College of Surgeons. Commission on Cancer Quality of Care Measures [cited 2018 June 27]. https://www.facs.org/quality-programs/cancer/ncdb/qualitymeasures

9. Pinkney TD, Calvert M, Bartlett DC, et al. Impact of wound edge protection devices on surgical site infection after laparotomy: multicentre randomised controlled trial (ROSSINI Trial). *BMJ* 2013;347:f4305.

10. Bhangu A, Kolias AG, Pinkney T, et al. Surgical research collaboratives in the UK. *Lancet* 2013 Sep 28;382(9898):1091-2.

11. Dindo D, Demartines N, Clavien P-A. Classification of Surgical Complications: A New Proposal With Evaluation in a Cohort of 6336 Patients and Results of a Survey. *Ann Surg* 2004;240(2):205-213.

12. Harris PA, Taylor R, Thielke R, et al. Research electronic data capture (REDCap) - a metadata-driven methodology and workflow process for providing translational research informatics support. *J Biomed Inform* 2009;42:377–81.

13. National Research Council. Recognising contributions to work in research collaboratives: Guidelines for standardising reporting of authorship in collaborative research. *Int J Surg* 2018;52:355.

14. United Nations Development Programme (UNDP). Human Development Data (1990-2015) [cited 2018 June 27]. hdr.undp.org/en/statistics

For peer review only - http://bmjopen.bmj.com/site/about/guidelines.xhtml

15. National Institute for Clinical Excellence Guidance and guidelines. Early and locally advanced breast cancer: Diagnosis and treatment [cited 2018 June 27]. https://www.nice.org.uk/guidance/cg80

16. Moran MS, Schnitt SJ, Giuliano AE, et al. SSO-ASTRO Consensus Guideline on Margins for Breast-Conserving Surgery with Whole Breast Irradiation in Stage I and II Invasive Breast Cancer. *Int J Radiat Oncol Biol Phys* 2014;88(3):553–64.

17. National Institute for Clinical Excellence Guidance and guidelines. Oesophago-gastric cancer: assessment and management in adults [cited 2018 June 27]. https://www.nice.org.uk/guidance/NG83

18. National Institute for Clinical Excellence Guidance and guidelines. Colorectal cancer: diagnosis and management [cited 2018 June 27]. https://www.nice.org.uk/guidance/cg131

19. Khatri C, Chapman SJ, Glasbey J, *et al.* Social media and internet driven study recruitment: evaluating a new model for promoting collaborator engagement and participation. *PLoS One* 2015;10:e0118899.

20. Alkire BC, Shrime MG, Dare AJ, et al. The Global Economic Consequences of Selected Surgical Diseases: A Modelling Study. *Lancet Glob Health*. 2015;3(Suppl 2):S21-S27.

For peer review only - http://bmjopen.bmj.com/site/about/guidelines.xhtml

AUTHORS CONTRIBUTIONS

All authors within the NIHR Global Health Research Unit on Global Surgery contributed to the design, drafting and review of this study protocol. In addition to this, Mr Ewen Harrison is also the overall guarantor for the article.

FULL AUTHORSHIP LIST

NIHR Global Health Research Unit on Global Surgery

Protocol development group: Stephen R Knight, Thomas M Drake, Dmitri Nepogodiev, J Edward F Fitzgerald, Adesoji O Ademuyiwa, Philip Alexander, Jean C Allen Ingabire, Sara W. Al-Saqqa, Bruce Biccard, Giuliano Borda, David Borowski, Sule Burger, Kathryn Chu, Damian Clarke, Ainhoa Costa, Justine Davies, Rachel Donaldson, Chikwendu Ede, O. James Garden, Dhruv Ghosh, James C. Glasbey, T Peter Kingham, Hosni Khairy Salem, Anyomih Theophilus Kojo, Zach Koto, Marie Carmela Lapitan, Ismail Lawani, Chiapo Lesetedi, Maria Lorena Aguilera, Charles Mabedi, Mayaba Maimbo, Laura Magill, Felix Makinde Alakaloko, Alex Makupe, Janet Martin, Antonio Ramos-De la Medina, Mark Monahan, Rachel Moore, Vanessa Msosa, Soloman Mulira, Alphonse Zeta Mutabazi, Elmi Muller, Joseph Musowoyo, Adewale Oluseye Adisa, Jean Léon Olory-Togbe, Riinu Ots, Ahmad Uzair Qureshi, Sarah Rayne, Tracey Roberts, Marie Dione Parreno-Sacdalan, Catherine Shaw, Neil Smart, Martin Smith, Richard Spence, Steph van Straten, Stephen Tabiri, Viki Tayler, Thomas G Weiser, John Windsor, Joseph Yorke, Raul Yepez, Richard Lilford, Dion Morton, Aneel Bhangu, Sudha Sundar, Ewen M. Harrison

For peer review only

Funding statement

This study was funded by Department for International Development (DFID) – Medical Research Council (MRC) - Wellcome Trust Joint Global Health Trial Development Grant (MR/N022114/1). A National Institute of Health Research (NIHR) Global Health Research Unit Grant (NIHR 17-0799) is supporting the establishment of surgical research units in a subset of contributing low-income countries. The views expressed are those of the authors and not necessarily those of the NHS, the NIHR or the UK Department of Health and Social Care.

Competing interests

None declared. GlobalSurg is run by the Surgical Research Gateway (SuRG) Foundation. The SuRG Foundation is a registered charity (charity number 1159898) whose object is to advanced the education of medical students and doctors in surgical science, clinical research and audit methods by promoting participation in collaborative clinical research and audit studies.

Patient consent

This study was reviewed by the South East Scotland Research Ethics committee and they have classified this as clinical audit/service evaluation in the UK. As such, this does not require specific consent to be obtained from patients.

Ethics approval

South East Scotland Research Ethics Service in Edinburgh

Word count: 2711

For peer review only - http://bmjopen.bmj.com/site/about/guidelines.xhtml

SUPPLEMENTARY FILE 1. Required data fields for all patients

**Patient characteristics**	
Patient ID	Local hospital field
Primary method of patient identification	Multidisciplinary team meeting / tumour board list, outpatient clinic list, theatre logbook, planned operating list, ward/handover list, staff memory
Age	Completed years
Gender	Male, Female, Unknown
Body mass index (weight (kg) / height^2 (metres))	Underweight (BMI <18.5)<br>Normal weight (BMI 18.5 to 24.9)<br>Overweight (BMI 25 to 30)<br>Obese (BMI >30)
Unintentional weight loss (≥10% over 6 months, include clothes size ref in key)	No, Yes, Unknown
Performance status	0, 1, 2, 3, 4, Unknown
ASA score	I, II, III, IV, V, Unknown
Smoking status	No-never, Stopped >6 weeks ago, Yes-current smoker, Unknown
Diabetes	No<br>Diet controlled<br>Medication (non-insulin) controlled<br>Insulin Controlled<br>Unknown
Human Immunodeficiency Virus (HIV) tested	No, Yes-NEGATIVE, Yes-POSITIVE
**Pathway**	
Presentation	Symptomatic, screening, detected incidentally, unknown
Date of first consult for cancer symptoms (may be estimated)	DD/MM/YYY
Who did the patient first consult for cancer symptoms?	Local clinic: family doctor / general practitioner<br>Local clinic: nurse<br>Local clinic: specialist doctor<br>Hospital: out-patient clinic<br>Hospital: in-patient<br>Other/non-medical/traditional healer<br>Unknown
Distance from home to hospital	< 10 km, 10-20 km, 20-50 km, 50-100 km, >100 km, Unknown
**Disease characteristics**	
Location	Breast, Gastric, Colorectal
Cancer specific information	*Fixed fields for each cancer (see specific cancer variables)*
Diagnosis (what tests were performed pre-operatively, please tick all that apply)	*Fixed fields for each cancer (see specific cancer variables)*
Clinical stage	TNM classification / Essential TNM Classification
Neoadjuvant therapy	*Fixed fields for each cancer (see specific cancer variables)*
**Operative characteristics**	
Date of admission	DD/MM/YY, 24 hour clock
Date and time of operation	DD/MM/YY, 24 hour clock
Urgency of operation	Elective, Emergency
Surgical intent (at completion of procedure)	Palliative, Curative
Was a surgical safety checklist used?	No-but available in this hospital, No-but available in this hospital, Yes, Unknown
Primary operation performed	*Fixed fields for each cancer (see specific cancer variables)*
**Pathology**	
Most valid basis for cancer diagnosis	Clinical only<br>Imaging<br>Exploratory surgery/endoscopy without histology<br>Tumour specific markers<br>Cytology<br>Histology of metastasis (secondary deposit)<br>Histology of primary
Histology	*Fixed fields for each cancer (see specific cancer variables)*
Size of invasive tumour	Centimetres

For peer review only

TNM (pathology)	
Number of INVOLVED lymph nodes in specimen	
TOTAL number of lymph nodes in specimen	
Histological grade	1, 2, 3, 4
Lymphatic or vascular invasion	No, Yes, Unknown
Resection margins	Fixed fields for each cancer (see specific cancer variables)
**Outcomes and adjuvant treatment**	
Length of postoperative stay	Continuous number of days
How was 30-day follow-up status achieved? (dropdown box)	Still an inpatient OR re-admitted<br>Clinic review<br>Telephone review<br>Community/home review<br>Discharged before 30 days and not contacted again
30-day mortality (if alive at the point of discharge and no follow-up information available, indicate Alive)	Alive, Dead (date of death), Unknown
30-day cancer-specific complications	Fixed fields for each cancer (see specific cancer variables)
30-day minor complication (CD I)	No, Yes, Unknown
30-day minor complication (CD II)	No, Yes, Unknown
30-day unexpected re-intervention (CD III)	No, Yes-NOT under general anaesthetic, Yes-under anaesthetic, Unknown
30-day unplanned critical care admission (CD IV)	No, Yes-single organ failure, Yes-multi organ failure, Unknown
30-day unplanned hospital readmission	No, Yes, Unknown
Surgical site infection	No<br>Yes, no treatment/wound opened only (CD I)<br>Yes, antibiotics only (CD II)<br>Yes, return to operating theatre (CD III)<br>Yes, requiring critical care admission (CD IV)<br>Yes, resulting in death (CD V)<br>Unknown
Post-operative haemorrhage	No<br>Yes, no intervention required (CD I)<br>Yes, drug treatment only (CD II)<br>Yes, intervention required (CD III)<br>Yes, critical care admission &/- intervention required (CD IV)<br>Yes, resulting in death (CD V)<br>Unknown
Planned adjuvant treatment	*Fixed fields for each cancer (see specific cancer variables)*

Those written in italics represent variables which have cancer-specific data points

For peer review only

SUPPLEMENTARY FILE 2. Required data fields for patients with breast cancer

**Disease characteristics**	
Diagnosis (what tests were performed pre-operatively, please tick all that apply)	> USS (No-not available, No-not indicated, No-indicated and facilities available, but patient not able to pay, Yes, Unknown)<br>> CT (No-not available, No-not indicated, No-indicated and facilities available, but patient not able to pay, Yes, Unknown)<br>> MRI (No-not available, No-not indicated, No-indicated and facilities available, but patient not able to pay, Yes, Unknown)<br>> Mammogram (No-not available, No-not indicated, No-indicated and facilities available, but patient not able to pay, Yes, Unknown)<br>> Fine needle aspiration (No-not available, No-not indicated, No-indicated and facilities available, but patient not able to pay, Yes, Unknown)<br>> Core biopsy (No-not available, No-not indicated, No-indicated and facilities available, but patient not able to pay, Yes, Unknown)<br>> Open/excision biopsy (No-not available, No-not indicated, No-indicated and facilities available, but patient not able to pay, Yes, Unknown)<br>> ER, PR, Ki-67, HER2 status assessed (No-not available in this hospital, No-but available in this hospital, Yes-NEGATIVE, Yes-POSITIVE, Unknown)
Stage (dropdown box)	TNM classification / Essential TNM classification<br>Unknown
Neoadjuvant chemotherapy	No, patient does not need it<br>No, patient needs it, but not available<br>No, patient needs it, facilities available, but patient not able to pay<br>No, planned but not given<br>Yes, NO anthracycline, NO taxane<br>Yes, anthracycline, NO taxane<br>Yes, anthracycline AND taxane<br>Yes, regimen unknown<br>Unknown
Neoadjuvant radiotherapy	No, patient does not need it<br>No, patient needs it, but not available<br>No, patient needs it, facilities available, but patient not able to pay<br>No, planned but not given<br>Yes (Cobalt)<br>Yes (Linear accelerator)<br>Yes (type unknown)<br>Unknown
Other neoadjuvant treatment (tick all that apply)	Hormone therapy<br>Biological therapy (HER2 inhibitor)<br>Oophrectomy<br>Other (free text)
**Operation**	
Primary operation	Mastectomy<br>Partial mastectomy / wide local excision / lumpectomy<br>Open biopsy of breast<br>Other operations on breast
Sentinel lymph node biopsy	No, not available in this hospital<br>No, but available in this hospital<br>Yes, single technique<br>Yes, dual technique<br>Unknown
Axillary lymph node biopsy	No, Yes, Unknown
Resection margins checked at time of surgery	No, not available in this hospital<br>No, but available in this hospital<br>Yes, by x-ray<br>Yes, by frozen section

For peer review only

	Unknown
Reconstruction	No, not available in this hospital<br>No, but available in this hospital<br>Yes, immediate – prosthesis<br>Yes, immediate – flap<br>Yes, planned at later stage
**Pathology**	
Histology	Invasive ductal carcinoma<br>Invasive lobular carcinoma<br>Ductal carcinoma in-situ (DCIS)<br>Other CANCER (specify)<br>Other BENIGN (specify)<br>Unknown, not available in this hospital<br>Unknown, but available in this hospital
Receptor status	ER, PR, Ki67, HER2<br>No-not available in this hospital, No-but available in this hospital, Yes-NEGATIVE, Yes-POSITIVE, Unknown
Resection margins	< 1 mm / tumour on inked margin<br>1-5 mm (NO tumour on inked margin)<br>>5 mm<br>Margins confirmed clear, but no distance given<br>Unknown, not available in this hospital<br>Unknown, but available in this hospital
**Outcomes and Adjuvant treatment**	
Post-operative seroma	No<br>Yes, no intervention/aspiration only (CD I)<br>Yes, antibiotic treatment only (CD II)<br>Yes, intervention required (CD III)<br>Yes, critical care admission &/- intervention (CD IV)<br>Yes, resulting in death (CD V)<br>Unknown
Planned adjuvant treatment (tick all that apply)	No, patient does not need it<br>No, patient needs it, but not available<br>No, patient needs it, facilities available, patient unable to pay<br>Yes, in this hospital<br>Yes, in another hospital in this country<br>Yes, in another hospital in a different country<br><br>Chemotherapy<br>Radiotherapy<br>Biological therapy (HER2 inhibitor)<br>Hormone therapy<br>Other (free text)

For peer review only

SUPPLEMENTARY FILE 3. Required data fields for patients with gastric cancer

**Disease characteristics**	
Diagnostic (what tests were performed pre-operatively, please tick all that apply)	> USS (No-not available, No-not indicated, No, indicated and facilities available, but patient not able to pay), Yes, Unknown)<br>> CT (No-not available, No-not indicated, No, indicated and facilities available, but patient not able to pay), Yes, Unknown)<br>> MRI (No-not available, No-not indicated, No, indicated and facilities available, but patient not able to pay), Yes, Unknown)<br>> Endoscopy (No-not available, No-not indicated, No, indicated and facilities available, but patient not able to pay), Yes, Unknown)<br>> Biopsy (No-not available, No-not indicated, No, indicated and facilities available, but patient not able to pay), Yes, Unknown)<br>> Staging laparoscopy (No-not available, No-not indicated, No, indicated and facilities available, but patient not able to pay), Yes, Unknown)
Stage (dropdown box)	TNM classification / Essential TNM classification
Neoadjuvant chemotherapy	No, patient does not need it<br>No, patient needs it, but not available<br>No, patient needs it, facilities available, but patient not able to pay<br>No, planned but not given<br>Yes<br>Unknown
Neoadjuvant radiotherapy	No, patient does not need it<br>No, patient needs it, but not available<br>No, patient needs it, facilities available, but patient not able to pay<br>No, planned but not given<br>Yes (Cobalt)<br>Yes (Linear accelerator)<br>Yes (type unknown)<br>Unknown
Other neoadjuvant treatment (tick all that apply)	Other (free text)
**Operation**	
Primary operation	Abdomen: Laparotomy with no other procedure<br>Abdomen: Diagnostic laparoscopy with no other procedure<br>Stomach: Total excision of stomach<br>Stomach: Partial excision of stomach<br>Stomach: Connection of stomach to jejunum<br>Stomach: Other open operations on stomach
Operative approach	Open<br>Laparoscopic (+/- open specimen extraction)<br>Laparoscopic converted to open<br>Robotic<br>Robotic converted to open
Site	Upper third (cardia/fundus)<br>Middle third (body)<br>Distal third (antrum/pylorus)<br>Entire stomach<br>Unknown
Cancer specific information	> Anastomosis: Not performed, handsewn, stapled, unknown<br>> D2 lymphadenectomy performed: No, Yes, Unknown<br>> Obstructed: No, Yes, Unknown<br>> Perforated: No, Yes, Unknown
**Pathology**	
Histology (dropdown box)	Adenocarcinoma<br>Lymphoma<br>Gastrointestinal stromal tumour (GIST)<br>Carcinoid<br>Other CANCER (specify)<br>Other BENIGN (specify)

For peer review only

	Unknown, histology not available in this hospital<br>Unknown, but histology available in this hospital
HER2 receptor status tested (on surgical resection specimen)	No-not available in this hospital, No-but available in this hospital, Yes-NEGATIVE, Yes-POSITIVE, Unknown
Resection margins	No residual disease (R0)<br>Microscopic residual disease (R1)<br>Macroscopic residual disease (R2)<br>Unknown, not available in this hospital<br>Unknown, but available in this hospital
**Outcomes and adjuvant treatment**	
Intra-abdominal abscess	No<br>Yes, no intervention (CD I)<br>Yes, antibiotics only (CD II)<br>Yes, surgical/radiological drainage (CD III)<br>Yes, critical care admission (CD IV)<br>Yes, resulting in death (CD V)<br>Unknown
Anastomotic leak	No<br>Yes, no intervention required (CD I)<br>Yes, drug treatment only (CD II)<br>Yes, intervention required (CD III)<br>Yes, critical care admission &/- intervention required (CD IV)<br>Yes, resulting in death (CD V)<br>Unknown
Planned adjuvant treatment (tick all that apply)	No, patient does not need it<br>No, patient needs it, but not available<br>No, patient needs it, facilities available, patient unable to pay<br>Yes, in this hospital<br>Yes, in another hospital in this country<br>Yes, in another hospital in a different country<br><br>Chemotherapy<br>Radiotherapy<br>Biological therapy (HER2 inhibitor)<br>Hormone therapy<br>HIPEC<br>Other (free text)

For peer review only

For peer review only

SUPPLEMENTARY FILE 4: Required data fields for patients with colorectal cancer

**Disease characteristics**	
Diagnostic (what tests were performed pre-operatively, please tick all that apply)	> USS (No-not available, No-not indicated, No-indicated and facilities available, but patient not able to pay, Yes, Unknown)<br>> CT (No-not available, No-not indicated, No-indicated and facilities available, but patient not able to pay, Yes, Unknown)<br>> MRI (No-not available, No-not indicated, No-indicated and facilities available, but patient not able to pay, Yes, Unknown)<br>> Endoscopy (No-not available, No-not indicated, No-indicated and facilities available, but patient not able to pay, Yes, Unknown)<br>> Biopsy (No-not available, No-not indicated, No-indicated and facilities available, but patient not able to pay, Yes, Unknown)<br>> Staging laparoscopy (No-not available, No-not indicated, No-indicated and facilities available, but patient not able to pay, Yes, Unknown)
Stage	TNM classification / Essential TNM classification<br>Unknown
Neoadjuvant chemotherapy	No, patient does not need it<br>No, patient needs it, but not available<br>No, patient needs it, facilities available, but patient not able to pay<br>No, planned but not given<br>Yes<br>Unknown
Neoadjuvant radiotherapy	No, patient does not need it<br>No, patient needs it, but not available<br>No, patient needs it, facilities available, but patient not able to pay<br>No, planned but not given<br>Yes (Cobalt)<br>Yes (Linear accelerator)<br>Yes (type unknown)<br>Unknown
Other neoadjuvant treatment (tick all that apply)	Other (free text)
**Operation**	
Primary operation	Abdomen: Laparotomy with no other procedure<br>Abdomen: Diagnostic laparoscopy with no other procedure<br>Small bowel: Formation of ileostomy only<br>Colon: Total excision of colon and rectum<br>Colon: Total excision of colon<br>Colon: Extended excision of right hemicolon<br>Colon: Excision of right hemicolon<br>Colon: Excision of transverse colon<br>Colon: Excision of left hemicolon<br>Colon: Excision of sigmoid colon<br>Colon: Other excision of colon<br>Colon: Formation of any colonic stoma<br>Colon: Other open operations on colon<br>Rectum: Abdominoperineal resection<br>Rectum: Resection with anastomosis of colon to anus<br>Rectum: Anterior resection with anastomosis<br>Rectum: Resection with closure of rectal stump (Hartmann's)<br>Rectum: Other open operations on rectum
Operative approach	Open<br>Laparoscopic (+/- open specimen extraction)<br>Laparoscopic converted to open<br>Robotic<br>Robotic converted to open
Cancer specific information	> Site: Caecum, Ascending colon, Transverse colon, Descending colon, Sigmoid colon, High rectum (>10 to 15cm from anal verge), Middle Rectum (>5 to 10cm), Low rectum (≤5cm), Unknown<br>> Anastomosis: Not performed, handsewn, stapled, unknown<br>> Obstructed: No, Yes, Unknown

For peer review only

	> Perforated: No, Yes, Unknown
Stoma formation	No,<br>Yes, loop ileostomy<br>Yes, end ileostomy<br>Yes, loop colostomy<br>Yes, end colostomy<br>Unknown
**Pathology**	
Histology (dropdown box)	Adenocarcinoma<br>Squamous cell carcinoma<br>Carcinoid<br>Lymphoma<br>Other CANCER (specify)<br>Other BENIGN (specify)<br>Unknown, histology not available in this hospital<br>Unknown, but histology available in this hospital
Perineural invasion	No, Yes, Unknown
Resection margins	No residual disease (R0)<br>Microscopic residual disease (R1)<br>Macroscopic residual disease (R2)<br>Unknown, not available in this hospital<br>Unknown, but available in this hospital
Circumferential margin (CRM)	Millimetres
**Outcomes and adjuvant treatment**	
Anastomotic leak	No<br>Yes, no intervention required<br>Yes, intervention required<br>Yes, critical care admission +/- intervention required<br>Unknown
Planned adjuvant treatment (tick all that apply)	No, patient does not need it<br>No, patient needs it, but not available<br>No, patient needs it, facilities available, patient unable to pay<br>Yes, in this hospital<br>Yes, in another hospital in this country<br>Yes, in another hospital in a different country<br><br>Chemotherapy<br>Radiotherapy<br>Biological therapy (HER2 inhibitor)<br>Hormone therapy<br>Liver resection (metastasis)<br>Lung resection (metastasis)<br>HIPEC<br>Other (free text)

For peer review only

South East Scotland Research Ethics Service

Waverley Gate
2-4 Waterloo Place
Edinburgh
EH1 3EG

NHS Lothian

Ewen M Harrison

Senior Lecturer in General Surgery /
Honorary Consultant Surgeon
NIHR Unit on Global Surgery (Universities of Birmingham, Edinburgh and Warwick)
Clinical Surgery
University of Edinburgh
Royal Infirmary of Edinburgh
Edinburgh
EH16 4SA

Date: 19/02/2018
Your Ref:
Our Ref: NR/161AB6
Enquiries to:
Direct Line: 0131 465 5679
Email:

Dear Mr Harrison,

Project Title: "GlobalSurg 3: Quality and outcomes in global cancer surgery: a prospective, international cohort study"

You have sought advice from the South East Scotland Research Ethics Service on the above project. This has been considered by the Scientific Officer and you are advised that, based on the email correspondence it does not need NHS ethical review under the terms of the Governance Arrangements for Research Ethics Committees (A Harmonised Edition).

If the project is considered to be health-related research you will require a sponsor and ethical approval as outlined in The Research Governance Framework for Health and Community Care. You may wish to contact your employer or professional body to arrange this. You may also require NHS management permission (R&D approval). You should contact the relevant NHS R&D departments to organise this.

For projects that are not research and will be conducted within the NHS you should contact the relevant local clinical governance team who will inform you of the relevant governance procedures required before the project commences.

This letter should not be interpreted as giving a form of ethical approval or any endorsement of the project, but it may be provided to a journal or other body as evidence that NHS ethical approval is not required. However, if you, your sponsor/funder feel that the project requires ethical review by an NHS REC, please write setting out your reasons and we will be pleased to consider further. You should retain a copy of this letter with your project file as evidence that you have sought advice from the South East Scotland Research Ethics Service.

Yours sincerely,

Helen Newbery
Scientific Officer
South East Scotland Research Ethics Service

Headquarters
Waverley Gate, 2-4 Waterloo Place
Edinburgh EH1 3EG
Chair: Mr Brian Houston
Chief Executive: Tim Davison
Lothian NHS Board is the common name of Lothian Health Board

For peer review only
For peer review only - http://bmjopen.bmj.com/site/about/guidelines.xhtml

South East Scotland Research Ethics Service

RESEARCH	SERVICE EVALUATION	CLINICAL / NON-FINANCIAL AUDIT	USUAL PRACTICE (in public health including health protection)
The attempt to derive generalisable or transferable new knowledge to answer questions with scientifically sound methods* including studies that aim to generate hypotheses as well as studies that aim to test them, in addition to simply descriptive studies.	Designed and conducted solely to define or judge current care.	Designed and conducted to produce information to inform delivery of best care.	Designed to investigate the health issues in a population in order to improve population health<br>Designed to investigate an outbreak or incident to help in disease control and prevention
Quantitative research – can be designed to test a hypothesis as in a randomised controlled trial or can simply be descriptive as in a postal survey.<br>Qualitative research – can be used to generate a hypothesis, usually identifies/explores themes.	Designed to answer: "What standard does this service achieve?"	Designed to answer: "Does this service reach a predetermined standard?"	Designed to answer: "What are the health issues in this population and how do we address them?"<br>Designed to answer: "What is the cause of this outbreak or incident and how do we manage it?"
Quantitative research - addresses clearly defined questions, aims and objectives.<br>Qualitative research – usually has clear aims and objectives but may not establish the exact questions to be asked until research is underway.	Measures current service without reference to a standard.	Measures against a standard.	Systematic, quantitative or qualitative methods may be used.
Quantitative research – may involve evaluating or comparing interventions, particularly new ones. However, some quantitative research such as descriptive surveys, do not involve interventions.<br>Qualitative research – seeks to understand better the perceptions and reasoning of people.	Involves an intervention in use only. The choice of treatment, care or services is that of the care professional and patient/service user according to guidance, professional standards and/or patient/ service user preference.	Involves an intervention in use only. The choice of treatment, care or services is that of the care professional and patient/service user according to guidance, professional standards and/or patient/service user preference.	Involves an intervention in use only. Any choice of intervention, treatment, care or services is based on best public health evidence or professional consensus.
Usually involves collecting data that are additional to those for routine care but may include data collected routinely. May involve treatments, samples or investigations additional to routine care. May involve data collected from interviews, focus groups and/or observation.	Usually involves analysis of existing data but may also include administration of interview(s) or questionnaire(s).	Usually involves analysis of existing data but may include administration of simple interview or questionnaire.	May involve analysis of existing routine data supplied under license/agreement or administration of interview or questionnaire to those in the population of interest. May also require evidence review.
Quantitative research – study design may involve allocating patients/service users/healthy volunteers to an intervention.<br>Qualitative research – does not usually involve allocating participants to an intervention.	No allocation to intervention: the care professional and patient/ service user have chosen intervention before service evaluation.	No allocation to intervention: the care professional and patient/service user have chosen intervention before audit.	No allocation to intervention.
May involve randomisation.	No randomisation.	No randomisation.	May involve randomisation but not for treatment/ care/ intervention.
Normally requires REC review but not always. Refer to http://hradecisiontools.org.uk/ethics/ for more information.	Does not require REC review.	Does not require REC review.	Does not require REC review.

Published October 2017 © Health Research Authority 2017. Copyright and other intellectual property rights in this material belong to the HRA and all rights are reserved. The HRA authorises UK healthcare organisations to reproduce this material for educational and non-commercial use.

Headquarters
Waverley Gate, 2-4 Waterloo Place
Edinburgh EH1 3EG
Chair: Mr Brian Houston
Chief Executive: Tim Davison
Lothian NHS Board is the common name of Lothian Health Board

For peer review only - http://bmjopen.bmj.com/site/about/guidelines.xhtml

South East Scotland Research Ethics Service

* UK Policy Framework for Health and Social Care Research definition of research:

> *"3.1 For the purpose of this policy framework, research is defined as the attempt to derive generalisable or transferable1 new2 knowledge to answer or refine relevant questions with scientifically sound methods3. This excludes audits of practice and service evaluations. It includes activities that are carried out in preparation for or as a consequence of the interventional part4 of the research, such as screening potential participants for eligibility, obtaining participants' consent and publishing results. It also includes noninterventional health and social care research (i.e. projects that do not involve any change in standard treatment, care or other services), projects that aim to generate hypotheses, methodological research and descriptive research. Projects whose primary purpose is educational to the researcher, either in obtaining an educational qualification or in otherwise acquiring research skills, but which also fall into the definition of research, are in scope of this policy framework. Activities that are not research according to this definition should not be presented as research and need not be conducted or managed in accordance with this framework. A decision tool that provides a definitive answer about whether a project counts as research under this policy framework is available at www.hradecisiontools.org.uk/research.*

---

1 NB This definition involves an attempt at generalisability or transferability, i.e. the project deliberately uses methods intended to achieve quantitative or qualitative findings that can be applied to settings or contexts other than those in which they were tested. The actual generalisability or transferability of some research findings may only become apparent once the project has been completed.

2 Including new knowledge about existing treatments or care.

3 Projects that are not designed well enough to meet this definition are not exempt from this policy framework – see paragraph 9.10.a.

4 This means the part of the research where a change in treatment, care or other services is made for the purpose of the research. It does not refer to other methodological 'interventions', e.g. issuing a postal survey.

Published October 2017 © Health Research Authority 2017. Copyright and other intellectual property rights in this material belong to the HRA and all rights are reserved. The HRA authorises UK healthcare organisations to reproduce this material for educational and non-commercial use.

Headquarters
Waverley Gate, 2-4 Waterloo Place
Edinburgh EH1 3EG
Chair: Mr Brian Houston
Chief Executive: Tim Davison
Lothian NHS Board is the common name of Lothian Health Board

For peer review only - http://bmjopen.bmj.com/site/about/guidelines.xhtml

STROBE 2007 (v4) Statement—Checklist of items that should be included in reports of *cohort studies*

Section/Topic	Item #	Recommendation	Reported on page #
**Title and abstract**	1	(*a*) Indicate the study's design with a commonly used term in the title or the abstract	1
		(*b*) Provide in the abstract an informative and balanced summary of what was done and what was found	3
**Introduction**			
Background/rationale	2	Explain the scientific background and rationale for the investigation being reported	5
Objectives	3	State specific objectives, including any prespecified hypotheses	6
**Methods**			
Study design	4	Present key elements of study design early in the paper	7-16
Setting	5	Describe the setting, locations, and relevant dates, including periods of recruitment, exposure, follow-up, and data collection	7-16
Participants	6	(*a*) Give the eligibility criteria, and the sources and methods of selection of participants. Describe methods of follow-up	8-16
		(*b*) For matched studies, give matching criteria and number of exposed and unexposed	Not applicable
Variables	7	Clearly define all outcomes, exposures, predictors, potential confounders, and effect modifiers. Give diagnostic criteria, if applicable	8-16
Data sources/ measurement	8*	For each variable of interest, give sources of data and details of methods of assessment (measurement). Describe comparability of assessment methods if there is more than one group	8-16
Bias	9	Describe any efforts to address potential sources of bias	14-16
Study size	10	Explain how the study size was arrived at	15
Quantitative variables	11	Explain how quantitative variables were handled in the analyses. If applicable, describe which groupings were chosen and why	14-16
Statistical methods	12	(*a*) Describe all statistical methods, including those used to control for confounding	14-16
		(*b*) Describe any methods used to examine subgroups and interactions	14-16
		(*c*) Explain how missing data were addressed	8
		(*d*) If applicable, explain how loss to follow-up was addressed	8, 13
		(*e*) Describe any sensitivity analyses	Not applicable
**Results**			

For peer review only

Participants	13*	(a) Report numbers of individuals at each stage of study—eg numbers potentially eligible, examined for eligibility, confirmed eligible, included in the study, completing follow-up, and analysed	Not applicable
		(b) Give reasons for non-participation at each stage	Not applicable
		(c) Consider use of a flow diagram	Not applicable
Descriptive data	14*	(a) Give characteristics of study participants (eg demographic, clinical, social) and information on exposures and potential confounders	Not applicable
		(b) Indicate number of participants with missing data for each variable of interest	Not applicable
		(c) Summarise follow-up time (eg, average and total amount)	Not applicable
Outcome data	15*	Report numbers of outcome events or summary measures over time	Not applicable
Main results	16	(*a*) Give unadjusted estimates and, if applicable, confounder-adjusted estimates and their precision (eg, 95% confidence interval). Make clear which confounders were adjusted for and why they were included	Not applicable
		(*b*) Report category boundaries when continuous variables were categorized	Not applicable
		(*c*) If relevant, consider translating estimates of relative risk into absolute risk for a meaningful time period	Not applicable
Other analyses	17	Report other analyses done—eg analyses of subgroups and interactions, and sensitivity analyses	Not applicable
**Discussion**			
Key results	18	Summarise key results with reference to study objectives	Not applicable
**Limitations**			
Interpretation	20	Give a cautious overall interpretation of results considering objectives, limitations, multiplicity of analyses, results from similar studies, and other relevant evidence	Not applicable
Generalisability	21	Discuss the generalisability (external validity) of the study results	Not applicable
**Other information**			
Funding	22	Give the source of funding and the role of the funders for the present study and, if applicable, for the original study on which the present article is based	1

*Give information separately for cases and controls in case-control studies and, if applicable, for exposed and unexposed groups in cohort and cross-sectional studies.

Note: An Explanation and Elaboration article discusses each checklist item and gives methodological background and published examples of transparent reporting. The STROBE checklist is best used in conjunction with this article (freely available on the Web sites of PLoS Medicine at http://www.plosmedicine.org/, Annals of Internal Medicine at http://www.annals.org/, and Epidemiology at http://www.epidem.com/). Information on the STROBE Initiative is available at www.strobe-statement.org.

For peer review only
